# Crossroads of Drug Abuse and HIV Infection: Neurotoxicity and CNS Reservoir

**DOI:** 10.3390/vaccines10020202

**Published:** 2022-01-27

**Authors:** Shilpa Sonti, Kratika Tyagi, Amit Pande, Rene Daniel, Adhikarimayum Lakhikumar Sharma, Mudit Tyagi

**Affiliations:** 1 Center for Translational Medicine, Thomas Jefferson University, 1020 Locust Street, Philadelphia, PA 19107, USA; sontis@chop.edu (S.S.); LakhikumarSharma.Adhikarimayum@jefferson.edu (A.L.S.); 2 Department of Biotechnology, Banasthali Vidyapith, Vanasthali, Jaipur 304022, Rajasthan, India; lsmst21030_kratika@banasthali.in; 3 Cell Culture Laboratory, ICAR-Directorate of Coldwater Fisheries Research, Bhimtal, Nainital 263136, Uttarakhand, India; amit.pande@icar.gov.in; 4 Farber Hospitalist Service, Department of Neurological Surgery, Thomas Jefferson University, Philadelphia, PA 19107, USA; Rene.Daniel@jefferson.edu

**Keywords:** neuroAIDS, HIV latency, drug abuse, antiretroviral therapy

## Abstract

Drug abuse is a common comorbidity in people infected with HIV. HIV-infected individuals who abuse drugs are a key population who frequently experience suboptimal outcomes along the HIV continuum of care. A modest proportion of HIV-infected individuals develop HIV-associated neurocognitive issues, the severity of which further increases with drug abuse. Moreover, the tendency of the virus to go into latency in certain cellular reservoirs again complicates the elimination of HIV and HIV-associated illnesses. Antiretroviral therapy (ART) successfully decreased the overall viral load in infected people, yet it does not effectively eliminate the virus from all latent reservoirs. Although ART increased the life expectancy of infected individuals, it showed inconsistent improvement in CNS functioning, thus decreasing the quality of life. Research efforts have been dedicated to identifying common mechanisms through which HIV and drug abuse lead to neurotoxicity and CNS dysfunction. Therefore, in order to develop an effective treatment regimen to treat neurocognitive and related symptoms in HIV-infected patients, it is crucial to understand the involved mechanisms of neurotoxicity. Eventually, those mechanisms could lead the way to design and develop novel therapeutic strategies addressing both CNS HIV reservoir and illicit drug use by HIV patients.

## 1. Introduction

If not treated, the human immunodeficiency virus (HIV) infection can result in the development of acquired immune deficiency syndrome (AIDS). HIV infection is still considered a global epidemic by WHO. As of 2018, approximately 37.9 million people are infected with HIV, and 770,000 people have died of HIV-associated comorbidity that year alone [1]. HIV is a retrovirus that selectively targets and kills the cells of the immune system that express CD4+ receptors, primarily T helper cells. Infection of HIV results in the severe deterioration of the immune system, which disposes the body to opportunistic infections and/or cancers. Moreover, in a small subset of infected cells, HIV enters a latent or silent state. The latent HIV reservoirs can persist undetected forever in infected persons as the complete reactivation of all the latent HIV in the system has never been achieved and seems impossible at this juncture. Hence, the main obstacle in the effort to achieve complete viral eradication is the existence of latent HIV proviruses. Extensive research has been performed to investigate the molecular mechanisms controlling HIV persistence and the establishment of HIV latency [2,3,4,5,6,7]. However, complete HIV eradication is not yet possible.

Fortunately, with the introduction of antiretroviral therapy (ART), the death rate due to HIV has reduced drastically. However, certain anatomical barriers allow the establishment of secluded HIV proliferation by hindering the optimal impact of ART regimens. Consequently, HIV sanctuaries are prevalent in certain anatomical sites, such as the central nervous system (CNS), lymphoid tissue, adipose tissue, and the gut-associated lymphoid tissue (GALT) [8]. Hence, besides purging the latent HIV pool, elimination of the HIV sanctuaries is also imperative for curing HIV infection. Fortunately, new ART regimens are quite efficient in eliminating HIV sanctuaries; however, the latent pool remains invincible. Recent evidence has shown that unintegrated HIV can also produce viral proteins, which exacerbate the host immune response and lead to excessive toxicity and cell death [9]. Hence, to make ART more effective, research efforts have been diverted to identify the mechanisms through which latent HIV can be reactivated before being targeted by antiretrovirals [9,10,11,12,13]. Latency reversal agents such as romidepsin, JQ-1, and panobinostat have effectively reactivated the peripheral latent viral reservoir. However, when evaluated in the CNS, agents such as bryostatin-1 and JQ-1 have been shown to contribute to the accumulation of amyloid-beta (Aβ) in astrocytes and cause neurocognitive impairment [14].

Neurocognitive impairment is an unresolved complication of HIV infection. HIV-associated neurocognitive disorder (HAND) manifests as either asymptomatic, mild, or severe neurocognitive impairment [15]. The most serious form of HAND is HIV-associated dementia (HAD), which manifests as severe dementia, concentration deficit, motor problems, and fluctuating behavioral alterations. Mild forms of HAND cause mild disruption of day-to-day activities. Assistance from family can greatly diminish any discomfort associated with this kind of HAND. Asymptomatic neurocognitive impairment (ANI) is the most common form of HAND, with mild cognitive impairment that does not interfere with daily activities but presents as a problem, nonetheless [16]. By virtue of effective suppression of viral replication, ART-taking individuals rarely acquire HAD. However, HAND is quite common among HIV patients, even those who are taking anti-HIV drugs religiously and keeping HIV levels below the detection limit [17]. Notably, there are some conflicting claims pertaining to the effects of antiretrovirals on CNS functioning, as a few studies also reported improvement following discontinuation of ART, possibly indicating neurotoxicity of the antiretrovirals [17,18,19]. The use of specific ART regimens or the physiology of selected individuals in those studies could be the reason behind this controversy. Nevertheless, the prevalence of mild neuropathies, such as HAND, is quite common even in regular ART-taking individuals.

Antiretroviral neurotoxicity, HIV persistence, transient HIV replication in the CNS, inflammation, mitochondrial dysfunction, and autophagy all contribute to the pathology of HAND [20,21]. Naturally occurring or acquired comorbid diseases and conditions cause further complications. Drug abuse is intimately linked with HIV infection and HAND. Many drugs of abuse cause neurotoxicity and increase the susceptibility of the CNS cells to HIV infection by upregulating genes or proteins necessary for HIV transcription/replication [22,23,24,25]. Drug abuse in the context of HIV infection is extremely complicated, with many unknown variables. The main objective of this review is to explore the mechanisms through which HIV infection and various drugs of abuse contribute to neurotoxicity that manifests as HAND. We will also discuss the possible outcome of certain ART regimens in contributing to HAND, especially in those HIV-infected people who use drugs (PWUDs).

## 2. HIV Epidemic among People Who Abuse Drugs

The impact of drug abuse on HIV progression is an ongoing investigation. There is increasing evidence that suggests the majority of HIV transmission in drug users occurs through sharing equipment used for injecting drugs, most often needles. Increasing numbers of outbreaks of HIV infection were reported in communities worldwide where injection is the primary route of drug administration [26,27,28,29]. A global systematic study identified that approximately 15.6 million people worldwide injected drugs as of 2015, and 17.8% of people who inject drugs (PWID) (~2.8 million people) are estimated to have been infected with HIV. While HIV prevalence among PWID varied substantially across geographical regions, eastern Europe and Latin America are reported to have the largest numbers of PWID living with HIV [30]. According to the CDC, approximately 11% of new HIV diagnoses in the United States occur among people with a history of injection drug abuse [31]. The introduction of needle and syringe programs that provide people with sterile needles has dramatically decreased the incidence of HIV among PWID [32]. Despite these efforts to treat HIV, reports of recent HIV outbreaks warrant the need for expanded efforts to prevent HIV transmission among PWID.

While risky sexual behavior is the major contributor to the HIV epidemic, non-sexual methods such as smoking, sniffing, or snorting of commonly abused drugs also contribute to the post-exposure spread of HIV [33,34]. Studies that focused on the different types of drugs abused by people positive for HIV found that crack-cocaine, in particular, increased the risk of HIV infection progressing to AIDS [35]. A study by Siddiqui et al. showed a rapid decline of CD4+ cell count in people who used cocaine when compared with those who did not [36]. Although inhalation and insufflation were traditionally the main routes of cocaine administration, injection of cocaine is becoming a popular route of administration, leading to recent outbreaks of HIV infection in cities such as Luxembourg [37].

## 3. Current Perspectives on HIV Infection in PWUDs

It is well known that drug use is widespread among people infected with HIV. The preferred drug of abuse among infected individuals was surprisingly dependent on the abuser’s region of residence [38,39,40]. Additionally, the differences in the type of substance abused, the method of administration, and the fact that a majority of HIV-infected individuals can, at any time, test positive for more than one drug of abuse complicates our understanding of how substance abuse and HIV infection potentiate neurologic decline [41,42]. To strategize disease prevention, understanding important factors such as drug and HIV pathology, neurodegenerative disease diagnostic biomarkers, as well as drug use demographics is critical.

The commonly abused drugs worldwide are opioids, cannabinoids, cocaine, methamphetamine, and alcohol. Public data files from the National Survey on Drug Use and Health (NSDUH) collected from 377,787 individuals (548 individuals infected with HIV) with diverse ethnicities, and socio-economic backgrounds were used to investigate the association between drug abuse and HIV infection. This cohort represents a nationally representative sample of adults in the United States. Approximately 80% of people infected with HIV reported drug abuse. Among the participants infected with HIV, 76.7% reported marijuana (cannabinoid) use, 57.6% reported cocaine use, and 11.4% reported heroin (opioid) use [43]. In the near future, an increase in marijuana abuse is a likely outcome in many communities in the United States due to the lobbying efforts to legalize it. To investigate the prevalence of drug abuse in a larger population, a cohort of 10,652 adults infected with HIV were evaluated and identified that 31% of the infected individuals abused marijuana, 19% abused alcohol, 13% abused methamphetamine, 11% abused cocaine, 4% abused opioids, and roughly 20% of the participants abused more than one drug [44]. A recent meta-analysis that analyzed 25 studies with more than 25,000 participants from various developed and developing countries showed that the pooled prevalence of alcohol use disorder among PLWHA (men and women) was 29.8%, with a higher prevalence in developed countries and men [45]. It is clear from these statistics that the prevalence of drug abuse in HIV communities is increasing every day. Massive research efforts are being attempted to understand the contribution of drug abuse to HIV disease progression as well as the role of ART amidst drug use and HIV progression. These topics are discussed in the subsequent sections.

## 4. The Role of ART on CNS (Dys)Function

Antiretroviral therapy (ART) has had a major impact on all aspects of HIV-1 infection, particularly on the CNS function. In the pre-ART era, CNS diseases were among the highest-ranked comorbidities associated with HIV infection. While ART has effectively reduced the HIV viral load and related comorbidities, the magnitude of its effect is variable and inconsistent [46]. Antiretroviral drugs commonly belong to four drug classes: nucleoside reverse transcriptase inhibitors (NRTIs), non-nucleoside reverse transcriptase inhibitors (NNRTIs), protease inhibitors (PIs), and integrase inhibitors (IIs). The most common ART regimen used today consists of two NRTIs and an integrase inhibitor [47]. NRTIs and NNRTIs are primarily reverse transcriptase inhibitors. NRTIs, however, cause off-target inhibition of mitochondrial DNA polymerase resulting in mitochondrial toxicity, energy depletion, and subsequent oxidative stress [48]. NRTIs are also known to induce ER stress and activate the UPR pathways in astrocytes *in vitro* [49]. This evidence suggests that NRTI-induced mitochondrial toxicity can indirectly cause neurotoxicity and contribute to HAND [50]. Efavirenz is the most notable NNRTI to be associated with CNS toxicity. Efavirenz has been considered as the most neurotoxic NNRTI demonstrating ER stress, mitochondrial toxicity, and decreased neuronal viability [51,52]. Protease inhibitors inhibit the protease enzymes required to cleave and release the mature viral proteins. Protease inhibitors exhibit a high degree of drug interactions and off-target effects that limit their use in ART [53]. Several studies attempted to investigate neurotoxic effects of PIs, albeit with mixed results; there is little evidence in support of PIs being neurotoxic [47]. Integrase inhibitors are the most effective antiretrovirals and are well-tolerated. They have fewer side effects associated with them, most of which are systemic [54]. However, a common neuropsychiatric side effect, insomnia, was often reported in II clinical trials [55]. Several *in vitro* studies have investigated and failed to identify underlying mechanisms for neurotoxicity suggesting other than a few neuropsychiatric symptoms, IIs are not associated with significant neurotoxicity [47]. Although some antiretroviral drugs are associated with neurotoxicity, several newer drugs are being evaluated for safety in ongoing clinical trials. Despite the efficacy of ART, neurocognitive symptoms persist in HIV patients suggesting a complex multifactorial association between antiretrovirals, HIV, and any other drug the patient might use.

## 5. Mechanisms of HIV-Dependent Neurodegeneration

HIV rapidly spreads into the CNS following infection and instigates the process of neuronal damage, eventually leading to HAND, even in the presence of effective ART. A significant association was established between severe neuropathies and AIDS-related death in the absence of effective ART [56]. The introduction of ART drastically decreased the plasma viral load and controlled peripheral viral replication but failed to protect from mild cognitive impairments associated with HAND [57].

Upon reaching the brain, HIV primarily targets resident macrophages, such as perivascular macrophages and microglia [58]. The frontal cortex, substantia nigra, and cerebellum are the primary targets of HIV, where extensive neurological damage can be seen upon infection [59,60,61]. Decreased synaptic and dendritic density and the presence of giant multinucleated cells as identified in postmortem brain tissues of HIV-infected people are the hallmark features of HAND. Current diagnostic markers indicating the presence of HAND include increased numbers of microglia with elevated tumor necrosis factor (TNF)-α mRNA and the presence of excitatory neurotoxins in CSF and serum [62].

### 5.1. CNS as HIV Reservoir

Purging HIV from all cells is the first step toward its eradication. However, a reservoir encases the virus and prevents its escape, thus posing as a barrier for eradication. According to Eisele and Siliciano, a true cellular reservoir should satisfy the following three criteria: (i) viral DNA must be integrated into the host cell genome; (ii) should be capable of harboring the virus in a dormant and non-infectious state for a long period; and (iii) possess the ability to produce fully activated virions upon stimulation [63]. HIV is capable of infecting and integrating into various CNS cell types (macrophages, microglia, and astrocytes). The long life span of these cells, the limited accessibility to ART, and the ability to harbor integrating viruses make them potential reservoirs, as they satisfy at least two out of the three criteria required to be considered as potential reservoirs [64]. However, it is challenging to determine if these cells can release replication-competent viruses as they reside in deep tissues that are inaccessible in living subjects. Evidence in support of CNS cells as HIV reservoirs comes from autopsied brains and *in vitro* studies that reported the presence of HIV in several CNS cell types [65,66,67], often evolving into a distinct genetic clade in these cells over time, even when the patients were on ART prior to death [68,69,70]. The viral persistence in these cells may be responsible for causing neurocognitive deficits and HAND.

### 5.2. General Mechanisms of HIV-Mediated Neurotoxicity

**Figure 1:**
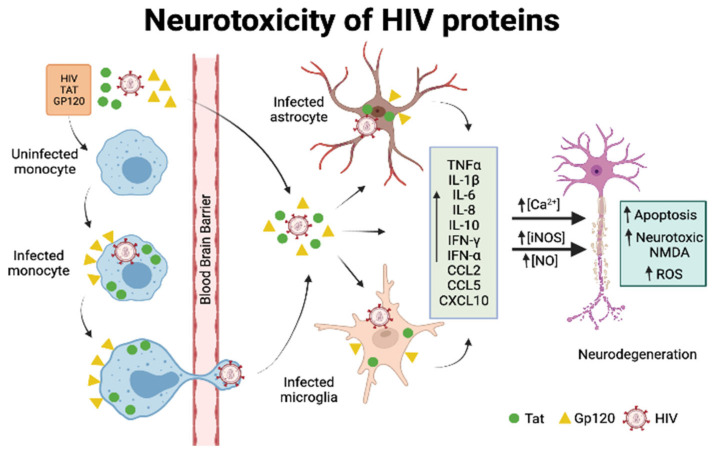
Neurotoxicity of HIV proteins. HIV proteins Tat and Gp120 cross the blood-brain barrier either directly or via infected monocytes and subsequently contribute to neurodegeneration either directly or indirectly by infecting glial cells and regulating the proinflammatory gene expression. The figure was created using BioRender software.

While neurodegeneration is a hallmark feature of HAND, the virus does not infect the neurons per se. Two general hypotheses can explain the initiation of neuronal damage by HIV. One of the mechanisms is the “direct injury,” where various viral proteins initiate neuronal injury and death. There is strong evidence in support of direct injury through viral envelope protein gp120 (the envelop glycoprotein) and Tat (transactivator of transcription), both of which show toxicity in CNS cells *in vitro* [71,72,73]. These proteins are well characterized, and a large amount of literature has been published describing the mechanisms through which different viral proteins mediate neurotoxicity [74] (Figure 1). On the other hand, the host macrophages and microglia are activated upon the detection of HIV and release various factors, either through interaction with viral proteins or by immune stimulation, that contribute to neurodegeneration. Some of the common mechanisms through which activated microglia trigger neuronal apoptosis involves Ca^2+^ overload; activation of p38 MAPK and p53; activation of cell cycle proteins and caspases; free radical formation; lipid release and peroxidation; and chromatin condensation [75,76,77,78,79] (Figure 1). This “indirect effect” is thought to be the predominant mechanism through which neuronal damage occurs [80,81].

### 5.3. Neurotoxicity of Viral Proteins gp120 and Tat

#### 5.3.1. Gp120

Gp120 is shown to be toxic in cultured dopamine neurons, causing a decrease in the size of the dendritic tree as well as the ability to transport dopamine [82]. Subsequent research on gp120 revealed multiple mechanisms of neurotoxicity. *In vitro* and *in vivo* administration of gp120 has been shown to induce apoptosis [83,84]. Several studies showed that gp120 disrupts the integrity of the mitochondrial membrane leading to the release of cytochrome c and activation of caspases, resulting in apoptosis [74]. A fair proportion of toxins released by gp120 from infected macrophages seem to target the ionotropic glutamate NMDAR (N-methyl-d-aspartate-type receptors) in the brain [85]. Indeed, *in vitro*, NMDAR antagonists have been shown to prevent HIV-associated neuronal cell death [86,87]. Under normal physiological conditions, activation of these receptors plays an important role in neurocognitive function. However, sustained activation of these receptors leads to excitotoxicity mediated by elevated intracellular calcium concentration, which subsequently leads to mitochondrial injury and dysregulation of cellular metabolism, resulting in the production of toxic free radicals [80,88]. In HIV infection, the activation of NMDAR receptors is sustained but mild, this state is indicative of neuronal dysfunction, and the neurons eventually undergo apoptosis [89]. Gp120 is also known to activate the hypothalamic-pituitary-adrenal (HPA) axis and increase the release of stress hormones, not only through NMDAR stimulation but also through the nitric oxide synthesis (NOS) pathway [90]. Gp120 strongly induced neuronal apoptosis by regulating the cellular sphingomyelin levels through the CXCR4-NADPH oxidase-superoxide-neutral sphingomyelinase-ceramide pathway [91].

#### 5.3.2. Tat

Tat is a viral regulatory protein, 86–101 amino acids in length, secreted by HIV-infected cells. The primary role of Tat is to recognize the 5′ TAR element in the HIV-1 RNA and recruit the host elongation factor p-TEFb (positive transcription elongation factor b) to HIV LTR promoter [92,93,94,95]. Tat also directly interacts with the histone acetyltransferase p300 and with the closely related CREB-binding protein (CBP) both *in vitro* and *in vivo*, and it targets these proteins to the integrated LTR promoter [96,97]. In a recent review, we have outlined the various mechanisms through which viral proteins modulate HIV latency in CNS cells [9]. CNS tissue damage and neuroinflammation in the presence of Tat may lead to the development of HAND [98].

The first report demonstrating the neurotoxicity of Tat identified Tat 31–61 as the reactive epitope that elevated intracellular calcium levels via activation of NMDAR and caused a dose-dependent increase in cytotoxicity in cultured human fetal brain cells [99]. While it has been shown that Tat mediates excitotoxicity via activating the NMDAR [100], further investigation revealed that Tat produced by HIV-1 subtype B and subtype C can directly bind to NMDAR, but Tat from subtype B was more neurotoxic. This underscores the importance of differences in HIV-1 subtypes and is consistent with the observation that HIV-1-associated neurocognitive impairment is more severe in regions where HIV-1-subtype B is prevalent [101].

Tat can cross the blood-brain barrier (BBB) and can be transported within the CNS, suggesting that sites of neuronal injury can vary distinctly from the site of actual viral infection [102]. The uptake of Tat by uninfected cells results in deleterious events, including abnormal cytokine secretion, altered gene transcription, NMDAR activation, and the initiation of apoptotic cascades [103,104]. It is well established that glutamate receptors are involved in the process of Tat-mediated neuronal cell death [99]. Further, NMDAR function may be modulated by dopamine D1-like receptors, and indeed D1-mediated pathways have been implicated in the mechanism of Tat-induced neurotoxicity [105,106,107]. Recent research showed that HIV-1 Tat-inhibited dopamine uptake and dopamine-transporter-specific ligand binding *in vitro* [108,109]. Inhibition of dopamine reuptake via Tat exposure in presynaptic dopaminergic neurons can influence the D1/NMDAR interaction in the postsynaptic neuron and subsequently trigger the NMDA receptor-controlled apoptotic cascade. Alternatively, pro-apoptotic D1-controlled signaling may be facilitated with the activation of NMDA receptors in a D1-expressing neuron when exposed to Tat [107]. This suggests that the mechanisms of HIV-1-associated neurodegeneration are similar to those involved in other neurodegenerative diseases and involve the dysregulation of glutamatergic and dopaminergic signaling pathways and excitotoxicity [107]. Other mechanisms for Tat neurotoxicity include increased oxidative stress [110], altered calcium homeostasis [111,112], stimulation of TNF-α and NF-κB [113], and activation of nitric oxide synthase and stimulation of nitric oxide production [114].

## 6. Neurotoxic Mechanisms of Drugs of Abuse

**Figure 2:**
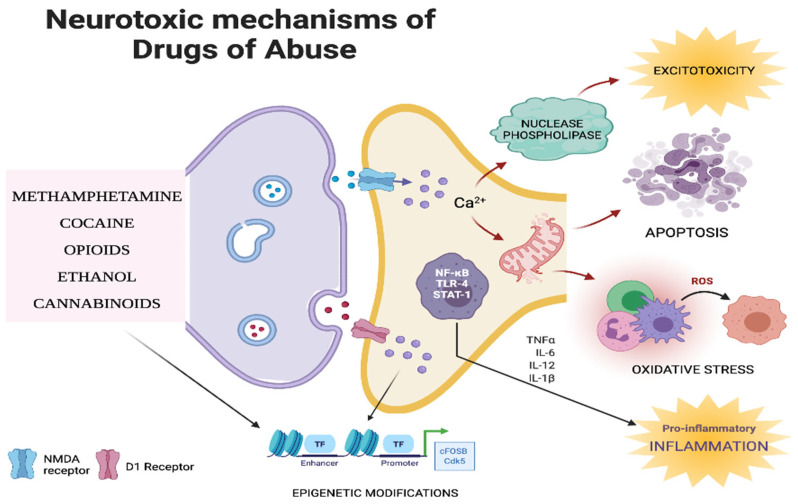
Outline of neurotoxic mechanisms of various drugs of abuse. Various drugs of abuse over-activate the glutamatergic and/or dopaminergic signaling pathways and initiate molecular cascades leading to excitotoxicity, apoptosis, oxidative stress, and inflammation. The figure was created using BioRender software.

Drug abuse causes substantial neurotoxicity that arises primarily due to the dysregulation of major neurotransmitter systems, mainly the dopaminergic and glutamatergic systems (Figure 2). Several drug-related, individual-related, and environmental factors influence the severity of drug-induced neurotoxicity [115]. The investigation led by several research teams identified key molecular mechanisms mediating drug-related neurotoxicity (Figure 2), which are expanded in the succeeding sections.

### 6.1. Inflammation

The innate and adaptive immune systems are major regulators of inflammatory responses. These systems are activated upon receiving damaged signals via pathogen-associated molecular patterns (PAMPs) and endogenous, danger-associated molecular patterns (DAMPs) that are detected by pattern recognition receptors (PRR) [116]. The response to signals initiated by PAMPs and DAMPs in the brain is generated by tissue-specific macrophages such as microglia and, more recently, astrocytes [117,118]. Upon recognition of these signals, PRRs release several cytokines having both pro-and anti-inflammatory properties. A balance between pro-and anti-inflammatory components is essential to maintain cellular homeostasis. Prolonged inflammation caused by the presence of elevated proinflammatory cytokines can lead to tissue damage and result in neuropsychiatric diseases [119]. Although less is known about the dysregulation of the neuroimmune system in substance abuse disorders, inflammatory changes are known to affect dopamine and glutamatergic systems, which are the key neurotransmitter systems involved in drug addiction and relapse [120,121].

### 6.2. Oxidative Stress

An imbalance between the ability of the cell to produce reactive oxygen species (ROS) and its capability to detoxify the reactive metabolites results in the building up of oxidative stress, which eventually leads to substantial cellular damage [122]. Overproduction of ROS, such as peroxides and free radicals, alters the normal redox state of the cell and can have devastating consequences. Their toxicity can affect many components of the cell, including proteins, lipids, and even nucleic acids [123]. Moreover, certain ROS are known to induce the expression of genes involved in signal transduction, and a few others function as cellular messengers in redox signaling. Alteration in their levels can disrupt normal cellular signaling [124].

The majority of drugs of abuse target the dopaminergic system and result in the accumulation of dopamine at the synapse, either by competing with the dopamine transporter (DAT) or by decreasing the reuptake of dopamine into the presynaptic neurons [125]. Early studies have established the toxicity of dopamine using both *in vitro* and *in vivo* experimental models [126]. Enzymatic and non-enzymatic metabolism of dopamine and/or related substances may result in the production of free radicals; drugs of abuse that act via the dopaminergic system, such as amphetamine, amphetamine derivatives, cocaine, 3,4-methylenedioxymethamphetamine (MDMA), and opioids, have all been reported to generate oxidative stress through similar mechanisms [127,128]. In addition to directly invoking oxidative stress through the generation of superoxide free radicals by the inhibition of catalase activity, cocaine also contributes to oxidative stress indirectly by decreasing the level of antioxidants such as glutathione and tocopherol (Vitamin E) [129,130,131]. In contrast, reports from mouse studies have shown that opioid derivatives target the antioxidant defense system, comprising the enzymes superoxide dismutase (SOD), catalase, and glutathione peroxidase (GPx) [132]. Heroin abuse exclusively caused an increase in DA oxidative metabolism, which resulted in DNA damage, protein oxidation, and lipid peroxidation [133].

### 6.3. Apoptosis

Apoptosis or programmed cell death is a highly regulated and controlled process that involves the genetically determined elimination of cells. Before undergoing apoptosis, cells exhibit several energy-dependent biochemical changes: the cytoskeleton collapses, the nuclear envelope disassembles, and the chromatin condenses and gets fragmented. The altered cell displays signals that are received by the neighboring macrophages, which rapidly phagocytose the cell and its contents [134]. Apoptosis is initiated through intrinsic or extrinsic pathways; the intrinsic pathway is activated when the cell recognizes internal stress and the extrinsic pathway invokes cell death through the recognition of signals exposed by surrounding cells [135]. In both cases, mitochondrial membranes of the cells are compromised, resulting in the activation of death receptors that in turn trigger the activation of caspases (proteolytic enzymes) [136]. Caspases are inherently present in the cell as inactive precursors (or procaspases) that are activated by adaptor proteins. Once activated, caspases initiate a proteolytic cascade where activated caspases cleave and activate other procaspases, which ultimately leads to proteolysis and cell death [135,136].

Apoptosis is necessary to ensure normal cell turnover and proper development as well as the functioning of the immune system. While cells normally undergo apoptosis when they are no longer needed, exposure to certain drugs of abuse can also initiate the apoptotic pathway. Amphetamines are known to stimulate mitochondrial pathways and induce apoptosis through several mechanisms involving p53, caspase activation, and the release of cytochrome c, leading to increased Bax/Bcl2 ratios [137]. Cocaine exposure activates the internal machinery involved in the apoptotic cascade without affecting the morphological characteristics of apoptotic cells [138,139]. Moreover, the oxidative stress generated by cocaine initiates apoptosis in neuronal progenitor cells [140]. Opioids and their derivatives also cause caspase activation and cytochrome c release from mitochondria, leading to apoptosis in animal and human models [141,142].

### 6.4. Excitotoxicity

Excessive stimulation of brain receptors by excitatory amino acids can induce neuronal death through the mechanism of excitotoxicity [143]. Excitotoxicity is a complex process associated with several pathologic and neuropsychiatric conditions. Presynaptic terminals in the CNS release neurotransmitters into the synaptic cleft, which bind and activate postsynaptic receptors of the target cell [144]. Upon activation by the neurotransmitter, ionotropic receptors facilitate the transport of ions, and if the ion favored is a cation, the receptor is classified as an excitatory receptor [145]. Calcium has an important role in maintaining multiple metabolic pathways. Increased intracellular calcium ion concentration causes activation of metabolic pathways; hence, their intracellular concentrations are maintained through ATP-coupled membrane transporters in the mitochondria and endoplasmic reticulum [146]. The excessive influx of calcium ions causes the activation of proteases, phospholipases, and endonucleases, all of which alter normal cellular functioning [147]. Excitatory amino acids such as glutamate were shown to release calcium following receptor depolarization [148]. The hyperstimulation of neuronal receptors by glutamate exacerbates the already high intracellular concentration and potentiates its harmful effects [143]. Disruption of the glutamate transport system or low energy production in the cells can cause glutamate to exert its excitotoxic potential [149].

Many drugs of abuse, including methamphetamine, cocaine, and opioids, cause excitotoxicity through excessive stimulation of NMDA receptors [128,150,151,152,153]. Alcohol abuse increases the activity of voltage-activated calcium channel and NMDA receptor activation and decreases GABA receptor activation, resulting in severe excitotoxicity. The stimulation of NMDA receptors by ethanol also results in the deficiency of thiamine, a vitamin important for several enzymatic reactions. The involvement of NMDA receptors in the pathogenesis of thiamine deficiency can probably explain why the symptoms of thiamine deficiency are similar to excitotoxicity [154].

### 6.5. Epigenetic Mechanisms

The non-genetic environmental factors that influence drug-related neurotoxicity suggest an important role for epigenetic mechanisms, which are a series of biological processes that cause permanent changes in gene expression without altering the DNA sequence. Epigenetics thus acts as a medium through which the environment interacts with the genome and influences health and disease [155]. While many epigenetic modifications are transient and temporary, some are stable. Random developmental events or behavioral experiences can cause permanent epigenetic changes in brain function. Such events can account for a person’s transition from a recreational drug user to a compulsive drug abuser [25,156,157]. A drug can directly bind to its specific molecular target and modulate downstream signaling cascades to alter gene expression and epigenetic mechanisms. Alternatively, a drug can indirectly cause epigenetic changes and alter gene expression by targeting the mesolimbic dopaminergic signaling pathway and downstream signaling cascades [156].

The majority of drugs of abuse target the dopaminergic pathway, which regulates the reinforcing activities important for survival [158,159]. Histone acetylation is a well-characterized epigenetic modification of the mesolimbic dopamine circuitry and is thought to be responsible for the reinforcing activity in response to psychostimulant exposure. Histone acetylation is witnessed in both acute and chronic exposure to psychostimulants [160,161]. Acute psychostimulant exposure causes the acetylation of histone 4 within the promoters of genes encoding transcriptional factors such as c-Fos and FosB, which are rapidly expressed in response to drug consumption [162].

Since virtually any pharmacological or genetic tool/intervention can cause epigenetic changes, it is worth questioning which epigenetic modification is responsible for or contributes to addiction in certain individuals. Epigenetic editing (or epigenome engineering) enables both precise manipulation of chromatin and studying the effects of such manipulation on gene expression and cell function. This novel technology has allowed targeted and specific rewriting of the epigenome [163]. Studies have used epigenetic editing in the brain to demonstrate that a single epigenetic modification at a single gene promoter can alter the expression levels of that gene [164]. An elegant study that served as a proof of concept of this new technology identified epigenetic modification at FosB and Cdk5 loci as the causal molecular changes that drive the pathogenesis of addictive behaviors in individuals who abuse cocaine [164,165].

### 6.6. Other Biochemical Mechanisms

Several other biochemical pathways contribute to neurodegeneration by drugs of abuse. These mechanisms are not as widely associated as the mechanisms. Hyperthermia is one such mechanism through which methamphetamine demonstrates neurotoxicity both in human and rodent models [166,167]. Biochemical changes, especially in the brain, are sensitive to temperature changes. Hyperthermia potentiates the depletion of dopamine and tyrosine hydroxylase by increasing oxidative stress [168]. Another mechanism associated with drug-related neurodegeneration is ER stress. ER stress is the first step in methamphetamine-mediated neurotoxicity, ultimately leading the cells to apoptosis. Methamphetamine-induced apoptosis involves the crosstalk between ER and mitochondria, which triggers both caspase-dependent and -independent death pathways [169]. Increased level of serotonin in neuronal synapses is also associated with drug-induced neurotoxicity. Accordingly, it was found that cocaine-induced serotonin accumulation compromises the BBB and induces hyperthermia in cocaine-using individuals [170,171,172,173].

## 7. Effect of Major Drugs of Abuse on HIV-Dependent Neurodegeneration

Opioids, cannabinoids (marijuana), cocaine, methamphetamine, and alcohol (ethanol) are among the most commonly abused drugs. Here we describe the effect of these major drugs of abuse on HIV-dependent neurodegeneration. Table 1 summarizes the key similarities and differences between the mechanisms of neurotoxicity of these major drugs of abuse.

### 7.1. Opioids

Approximately 2 million people in the United States abuse opioids in the form of heroin and/or prescription opioids [174]. Addiction to prescription opioids often leads to risky behaviors such as injection drug use: in fact, approximately 36% of new HIV cases in the United States are seen among people who inject opioids [175,176,177]. Similarly, 20–50% of people suffering from HIV are prescribed opioids and hence are more likely to develop opioid addiction [178]. In addition, people with HIV have an increased risk of death from long-term opioid abuse [179]. It is thus essential to identify the role of opioids in contributing to HIV infection and associated comorbidities.

Altered signal transduction via classical opioid receptors, μ (Mu; MOR), κ (Kappa; KOR), and δ (Delta; DOR), contribute to HIV-induced CNS damage. These receptors are expressed on the microglial, macrophage, and astrocytic cell types, all of which are capable of harboring HIV [180]. *In vitro* experiments demonstrate that several mechanisms mediate opioid-induced viral replication, such as microRNAs that target the degradation of HIV genes [181], increased expression of galectin-1 [182], inhibition of interferon, and increased expression of CCR5 [183]. Crosstalk between chemokine and opioid receptors enhances the neuropathogenesis caused by HIV. Opioid exposure to astrocytes infected with HIV exacerbated Tat-induced neuronal damage through the activation of CCR5 receptors. Interestingly, through pharmacological inhibition of CCR5 by the HIV entry inhibitor, maraviroc, morphine exerted a neuroprotective effect in response to Tat [184]. Opioid exposure to astrocytes decreased the expression of glutamate transporters such as GLT-1 and GLAST, and this disruption was further potentiated in the presence of HIV proteins [185,186]. The role of specialized potassium channels was recently discovered in opioid-induced glutamate released from astrocytes, possibly mediating through Gαi-coupled GPCR activity [187].

Opioids also tamper with the integrity of the BBB by increasing P-glycoprotein concentration in the brain, thus facilitating the entry of monocytes across the BBB [188,189]. Dopamine is a key player in opioid-induced euphoric effects. Circulating monocytes prefer to aggregate in brain regions with increased dopamine concentrations [190]. These monocytes express surface receptors that bind to dopamine, and upon binding, dopamine signaling is increased, probably due to the activity of ADAM17, a metalloproteinase [190,191]. Dopamine, in turn, increases viral entry into these monocytes and monocyte-derived macrophages, resulting in increased viral replication, indicating that dopamine release in response to opioids can cause an increase in HIV infection through circulating monocytes [192,193].

Opioid exposure is closely associated with increased oxidative stress. While acute morphine exposure to murine macrophages decreased nitric oxide production, chronic exposure increased the production of ROS through miRNA activation [181,194]. *In vivo* morphine administration in mice is also seen to increase ROS. In addition, Tat synergistically increases ROS with acute morphine exposure in murine microglia [195]. This evidence indicates that opioids contribute to HIV disease progression through oxidative stress in cells harboring HIV. Opioids also induce cytokine secretion that fuels neuroinflammation. A total of 24 h exposure of morphine to murine microglia upregulated the secretion of proinflammatory IL6, MCP-1, and TNFα, but this did not replicate in a human microglial model [195]. Further *in vitro* and *in vivo* studies are required to determine the effect of opioids on cytokine secretion in other CNS cell types infected with HIV.

Increased expression of regulatory T cells (Tregs) is seen in people addicted to opioids when compared to healthy controls [196]. Chronic opioid abuse is characterized by altered functions of MOR, KOR, and DOR in T cells, contributing to systemic immunosuppression and increased susceptibility to infection [197,198,199]. While in CD4+T cells, opioid receptor stimulation failed to reactivate the virus in latently infected cells, activity at the KOR and DOR was shown to inhibit HIV production [197,198]. The role of opioid exposure to T-cell activity during HIV infection in CNS cells is not clear.

Initiation of ART can further complicate neuropathogenesis. Most opioid drugs are metabolized by the cytochrome P family enzymes such as CYP3A4, CYP2D6, CYP2C19, CYP2C9, and CYP2D67 [200]. Many ART drugs inhibit cytochrome P enzymes and thereby increase the efficacy of opioids such as oxycodone [201]. On the other hand, certain antiretroviral drugs can also decrease the effects of opioids such as hydrocodone and cause symptoms of opioid withdrawal [202]. Future studies should thus aim to evaluate the combinatorial effect of opioid abuse, HIV infection, and ART on neuroinflammation in macrophages and microglia. This might unravel the molecular mechanisms through which these cells accelerate neurodegeneration in opioid abusers who are HIV-positive.

### 7.2. Cannabinoids

Cannabinoids rank third among the drugs abused in the United States, falling only behind alcohol and nicotine (tobacco) [203]. Cannabinoid consumption is a common practice among people living with HIV, who consume them for palliative and/or recreational purposes. Cannabis use disorder (CUD) is currently on the rise, and it is imperative to address the extent of its neurotoxicity in HIV-infected individuals in the context of finding a proper treatment regimen. The effects of marijuana, the major cannabinoid used and abused, are attributed to δ-9-tetrahydrocannabinol (THC), the main psychoactive ingredient in the cannabis plant. THC binds to cannabinoid CB1 receptors in the brain and modulates various neurological changes, including cognition [204]. A study conducted on 282 HIV-infected individuals who used marijuana showed a significant correlation between the consumption of marijuana during advanced stages of HIV infection and memory loss, but this correlation was absent in the uninfected or individuals in early stages of HIV infection, indicating synergy between the extent of HIV infection and marijuana use [205]. Excessive use of cannabinoids in HIV-infected individuals is associated with modified inflammatory and neurotoxic processes. The HIV envelop protein gp120 is known to degrade the synaptic network of hippocampal neurons through the activation of signal cascade via CXCR4 and release of IL-1β [206,207]. IL-1β further potentiates this synapse loss, which triggers the activation of NMDA receptors that control synaptic plasticity. This synapse loss is prevented by WIN55212-2, a full agonist of the cannabinoid receptors, through its action on CB2 receptors; however, in the presence of HIV Tat, WIN55212-2 does not affect synapse loss [206].

Endogenous compounds known as endocannabinoids act at the brain’s cannabinoid receptors, CB1 and CB2. Endocannabinoids have a neuroprotective effect in several neurodegenerative disorders and are being increasingly explored as immune modulators. Endocannabinoids are primarily anti-inflammatory mediators that suppress inflammation by inhibiting the release of inflammatory mediators [208]. Owing to their neuroprotective effects, it is important to document the changes in endocannabinoid signaling in response to HIV infection to devise strategies to enhance or suppress signaling by targeting the components of the endocannabinoid system.

CB1 and CB2 receptors are the most extensively studied and well-characterized components of the endocannabinoid system. Several reports indicate changes in CB1 and CB2 cannabinoid receptors during SIV and HIV infection. An increase in CB2 receptor expression is seen in peripheral microglial cells of rhesus macaques with SIV-induced encephalitis (SIVE) [209]. Upregulation of CB1 and CB2 receptors was seen in macrophage and microglia tissue isolated from patients with HIV encephalitis [210]. CB2 receptor activation by selective agonists is known to trigger an anti-inflammatory response [211]. AM1241, a full CB2 agonist, is shown to increase neurogenesis, as opposed to astrogenesis and gliogenesis, in the hippocampus of the GFAP/Gp120 transgenic mouse model [212]. Although CB1 receptors mediate neuroprotective functions [213,214], their involvement with HIV viral proteins is still under investigation. An increase in the enzyme fatty acid amide hydrolase (FAAH), which is responsible for the breakdown of endocannabinoids, is also seen in the astrocytes of SIV-infected macaques [209]. Increased FAAH levels indicate a direct association of FAAH in the anti-inflammatory response against SIV, and it is hypothesized that FAAH counteracts the overexpression of eicosanoids and TNF-α induced by the SIV-infected monocytes in SIVE [215,216]. Through the activation of FAAH, the HIV envelop protein gp120 decreases the levels of the endocannabinoid anandamide, which is a full agonist at the CB1 receptors and induces neuronal apoptosis of rat brain neocortex [217].

While CB2 receptors are considered to play a role in mitigating HAND and related neurodegenerative diseases, CB1 receptor activation mediates the psychoactive properties of exogenous cannabinoids that can become a serious adverse effect when used for the prolonged therapeutic benefit [218]. Cannabinoid-induced cognitive decline cannot be ignored, especially when implicated in the treatment of HAND [219]. Finally, the addictive properties of prolonged cannabinoid use or abuse limit its use to treat HAND [220]. Marijuana is a potent inducer of the CYP1A2 enzyme, and it does so through aromatic hydrocarbon receptor activation [221]. Both δ-9-tetrahydrocannabinol (THC) (a potent CB1 agonist) and cannabidiol (a potent CB2 agonist) are metabolized by various cytochrome P enzymes (CYP3A4 and CYP2C19), which also metabolize multiple ART drugs [222]. The competition between these two drug classes can increase the plasma concentration of ART, causing a heavy renal/hepatic demand and potentially proving to be toxic.

### 7.3. Cocaine

Cocaine is one of the most abused drugs that modulate CNS functions. Cocaine is a monoamine reuptake inhibitor that binds to monoamine transporters and inhibits the reuptake of extracellular dopamine, and to some extent, of serotonin and norepinephrine. All three neurotransmitters mediate reinforcing activities in the CNS, and by elevating their extracellular concentrations, cocaine thereby acts as a powerful psychostimulant [223]. Clinical evidence suggests decreased brain metabolic function with significant neuropsychological, behavioral, and neurocognitive disorders in HIV-infected patients who also abuse cocaine [224]. Recent studies have linked the effects of cocaine abuse and/or HIV infection with several intracellular molecules and signaling cascades within the cells of the CNS. Cocaine-induced neurotoxicity is also attributed to the modulation of CNS macrophages and astroglia functioning [225,226].

The stimulatory effect of cocaine has been shown to enhance HIV gene expression and replication, with an eventual impact on HIV-associated pathogenicity in the CNS cells, both *in vitro* and *in vivo* [227,228,229,230,231]. The biological interaction between cocaine and HIV interaction was studied in a severe combined immunodeficiency (SCID) mouse model with implanted human peripheral blood mononuclear cells and infected with an HIV reporter virus (huPBL-SCID). Cocaine administration in these models increased the expression of CCR5 and CXCR4 coreceptors, which are required for HIV entry and subsequent replication, via a sigma-1 receptor (σ-1R)-mediated mechanism. The implication of σ-1R in cocaine-mediated HIV pathogenicity was further confirmed when a σ-1R antagonist abolished the effects of cocaine on HIV-1 replication [232]. In dendritic cells, cocaine upregulated another HIV coreceptor, DC-SIGN (dendritic cell-specific intercellular adhesion molecule-3-grabbing non-integrin) through dysregulation of mitogen-activated protein kinases (MAPKs) [233].

Our group demonstrated the underlying molecular mechanism through which cocaine enhances HIV gene expression and subsequently its replication. We discovered that cocaine mobilizes NF-κB and p-TEFb by stimulating kinases such as ribosomal S6 kinase 1 (RSK1) and mitogen- and stress-activated kinase 1 (MSK1). Acute and chronic cocaine exposure to monocytic cell lines (THP1 and U937) as well as primary monocyte-derived macrophages (MDMs) increased the nuclear translocation of NF-κB and potentiated its ability to interact with histone acyltransferases (HATs). We showed that RSK1-induced NF-κB activation lasts longer in the presence of cocaine and contributes to the lingering stimulatory effects of cocaine [25,157]. The functional activity of NF-κB is determined as a measure of its interactions with HATs. Activation of MSK-1 phosphorylates the p65 subunit of NF-κB at serine 276 and histone H3 at serine 10. This epigenetic modification enhances the interaction of NF-ĸB with HATs, specifically P300, resulting in enhanced HIV transcriptional initiation. However, increased phosphorylation of histone H3 at serine 10 promotes the recruitment of positive transcription factor b (p-TEFb) to the HIV-1 LTR. p-TEFb is required for the elongation phase of HIV-1 transcription. Thus, cocaine promotes both HIV transcriptional initiation via NF-kB and transcriptional elongation through p-TEFb [23].

Cocaine is known to amplify the neurotoxic responses of HIV proteins Tat and gp120 *in vitro* by augmenting the oxidative stress caused by these proteins [234,235,236,237]. A combination of cocaine and gp120 in rat primary neurons demonstrated a synergistic increase in cell toxicity through the production of ROS and expression of the proapoptotic protein Bax [238]. Similarly, cocaine augmented Tat-mediated mitochondrial depolarization and production of ROS, leading to oxidative stress and neurotoxicity in primary rat hippocampal cultures [239]. Using a specific D1 dopamine receptor antagonist, it was shown that D1 dopamine receptors were involved in cocaine-mediated neurotoxicity in HIV-infected cells, as the toxicity of Tat was minimized upon blocking this receptor in hippocampal neuronal culture [108].

A recent investigation revealed that cocaine use by HIV-infected individuals induces epigenetic modifications, in the form of DNA methylation and hydroxymethylation, in the mitochondrial DNA of the brain. This causes mitochondrial dysfunction that gives rise to neuropathies, including HAND [240]. In addition, somatodendritic injury is often associated with neurologic impairment in patients with HAND. Consistent with this, exposure of primary hippocampal neurons to cocaine as well as gp120 resulted in the enhanced loss of neuronal dendrites, thus suggesting a possible mechanism for cocaine-mediated neurotoxicity [241]. Combined administration of intraperitoneal cocaine and intracerebral recombinant gp120 to wild-type Wistar rats resulted in increased iNOS expression and neuronal apoptosis in the neocortex, corroborating the evidence for cocaine-mediated neurotoxicity in HAND [242].

With the advent of NGS (next-generation sequencing), new pathways and molecular mechanisms underlying rare and known neurodegenerative pathologies are being discovered. RNA sequencing analysis in rat hippocampal neurons identified that several key genes involved in lipid and cholesterol metabolism such as sterol O-acyltransferase 1/acetyl-coenzyme A acyltransferase 1 (SOAT1/ACAT1), sortilin-related receptor L1 (SORL1), and low-density lipoprotein receptor-related protein 12 (LRP12) were disrupted when exposed to Tat and cocaine. These genes are implicated in the pathology of Alzheimer’s disease, thus opening up novel molecular targets involved in HIV- and cocaine-mediated neuronal dysfunction [243].

Cocaine is metabolized differently in males and females. Women can metabolize cocaine faster and hence can be more sensitive toward its effects [244]. The enzymes CYP3A4 and CYP3A5 partially metabolize cocaine and interfere with ART metabolism and bioavailability [245].

### 7.4. Methamphetamine

Methamphetamine is abused by more than 33 million people worldwide, and the numbers are still steadily rising [246]. A recent article in *The Lancet* emphasized the possible occurrence of the second wave of methamphetamine abuse, owing to the allocation of resources and government funds toward the stringent regulation of opioid abuse [247]. The effect of methamphetamine abuse on brain pathology has been well characterized by several studies. MDMA, or 3, 4 methylenedioxymethamphetamine (ecstasy), a derivative of methamphetamine, is a popular club drug often used in conjunction with methamphetamine and has a more profound effect on the brain than methamphetamine alone. These psychostimulants are known to increase risky sexual behavior among users and contribute to increased HIV transmission rates [248]. Hence, in the context of methamphetamine abuse in HIV-infected individuals, it is important to understand the neurotoxicity mechanisms of methamphetamine and its interaction with HIV proteins.

It is well established that methamphetamine can induce substantial neuronal damage to the CNS by disrupting the function of neurons and glia [249,250,251]. Methamphetamine targets the neurons directly, as well as indirectly, by disrupting the BBB or impairing glial signaling. Dopaminergic, serotonergic, and noradrenergic neurons are targeted by methamphetamine, with dopaminergic neurons being the most susceptible in the HIV-infected population [237,252]. The mechanisms through which methamphetamine exerts neurotoxicity have been well documented [253,254,255]. Briefly, methamphetamine exerts a destructive positive feedback cycle by inhibiting the function of vesicular monoamine transporter 2 (VMAT2) and the dopamine transporter (DAT), which cumulatively increase the production of dopamine and oxidative byproducts that lead to neuronal damage [249,256]. Exposure to methamphetamine can increase the levels of extracellular glutamate, which can cause excitotoxic neuronal injury, and over-activation can lead to the death of NMDA receptors [257,258].

Several studies report depletion of dopamine reserves in HIV-infected individuals, and the use of methamphetamine exacerbates this depletion [237,259]. HIV proteins, Tat and Gp120, are toxic to dopaminergic neurons [260]. Methamphetamine synergistically acts with HIV Tat to diminish dopamine levels [261]. There is evidence in support of Tat interacting with and inhibiting VMAT2 and DAT, the same molecular targets of methamphetamine, justifying the synergy between methamphetamine and HIV proteins [262,263]. It has been recently shown that crosstalk occurs between neurons and microglia that influences HIV infectivity and subsequent inflammation or neuronal damage. Using a neuron-hμglia (human microglia) co-culture, it has been demonstrated that HIV-infected microglia are the main drivers of neurotoxicity. Methamphetamine abuse further potentiates this neurotoxicity via activation of the sigma-1 receptor (σ-1R) in the microglia [264].

Methamphetamine is metabolized by the enzyme CYP2D6 through N-dealkylation. Inhibition of CYP2D6 by ART drugs can increase the bioavailability of methamphetamine and related drugs, thereby exacerbating neurotoxicity [265]. Methamphetamine is also known to interact with CYP3A4 and, as a result, can modulate the bioavailability of PI ART drugs [266].

### 7.5. Ethanol

Ethanol-associated neurodegeneration involves neuronal apoptosis, among other mechanisms. Reports of ethanol-induced apoptosis have been documented in well-characterized rodent brain models [267,268,269]. Ethanol suppresses neuronal activity by altering glutamate and GABA transmission. Chronic alcohol consumption has been known to alter various functions of the immune system, including both humoral and cell-mediated processes.

There is much controversy as to whether ethanol serves as a cofactor in AIDS development [270,271]. Ethanol exacerbated opportunistic infections in a murine AIDS-like syndrome model and contributed to the progression of AIDS [272,273,274]. Ethanol is known to have both direct and indirect effects on HIV replication and expression *in vitro*. It has been suggested that ethanol stimulates HIV replication in latently infected cells by altering cytokines that increase HIV-1 expression [275]. In Jurkat T cells, ethanol increased tumor necrosis factor α (TNF-α)-stimulated HIV transcription [276]. Exposure of ethanol and its metabolites, acetaldehyde, and acetate, to a human macrophage cell line resulted in the diminished production of interleukin 1alpha (IL-1alpha), IL-1beta, and intracellular cytokine. Ethanol has been shown to decrease macrophage function during HIV-1 infection [277]. Ethanol also induced direct cell death in human neurons at a clinically relevant dose range, and at low and moderate concentrations, it strongly potentiated HIV-1 gp120-induced neuronal apoptosis via both the death receptor and NMDA receptor pathways [278]. Similarly, in the presence of ethanol, the HIV-1 regulatory protein Tat synergistically increased neuronal apoptosis [275] and neutrophil dysfunction in transgenic mice [279]. Co-exposure of mice to intraperitoneal ethanol and Tat resulted in increased production of oxidative stress and proinflammatory cytokines (IL-1B, MCP-1, and TNFα) as well as elevation of ICAM-1 mRNA expression.

Ethanol is largely metabolized by the cytochrome enzymes CYP2E1 and CYP3A4, which also metabolize the majority of ART drugs [200]. As with other drugs of abuse, this competition for the same enzymes can modulate each drug class’s bioavailability and increase their respective toxicities.

## 8. ART, Drugs of Abuse, and Hand

The main approach implemented to prevent HIV incidence in PWID/PWUDs is through ART. Peripheral viral loads have been very effectively suppressed by ART. However, the benefits of reduced viral load and health improvement of HIV patients come at the cost of toxic side effects of the drugs used in the ART regimen, primarily due to their continuous use under all physiological situations during their lifetime. In some HIV cohorts, antiretrovirals with a greater CNS penetration (CPE) score improved viral suppression in the CNS while still exhibiting impaired neurocognitive performance; in other HIV cohorts, ART with a high CPE score showed improved neurocognitive performance when it consisted of three or more antiretrovirals, probably because these patients may require more than three antiretrovirals for a sufficient amount of the drug to reach the brain and treat HIV in the nervous system [280,281].

Drug abuse is widely prevalent all over the United States, especially among the HIV-infected population. Before the introduction of ART, some studies reported an independent association between drug abuse and progression of AIDS and mortality [282]. However, with the widespread use of ART, it has become increasingly difficult to separate the side effects of ART medication in active drug users from the physiologic effects of drug abuse [283]. Several reports suggest a strong correlation between drug abuse and poor ART adherence and that drug abuse, even intermittently, can cause decreased ART adherence [284,285]. This decreased ART adherence in PWUDs can partly be explained as a consequence of drug interactions between antiretrovirals and drugs of abuse. Enzymes belonging to the cytochrome P (CYP) family are responsible for metabolizing the drugs of abuse as well as most xenobiotics, including antiretrovirals [265,286,287,288]. The competition between antiretrovirals and drugs of abuse modulates each other’s bioavailability, thus providing a possible explanation for persistent disease progression in PWUDs while on ART regimen [289]. However, recently it has been shown that drug abuse can aggravate disease progression independent of ART adherence [290], and accelerated disease progression often leads to NeuroAIDS, especially in PWUDs [291,292]. Approximately half of the HIV-infected PWUDs have an underlying mental illness, which can potentiate the risk of overdose or behavioral conditions when left untreated. Active drug abuse triggers the release of proinflammatory cytokines and chemokines from CNS cells, resulting in neuroinflammation [293,294]. Drug abuse also compromises the integrity of the BBB, which leads to increased migration of HIV-infected monocytes into the CNS [295].

Currently, no FDA-approved pharmacotherapy exists to treat addiction in HIV-infected individuals, especially those who use more than one illicit drug [296]. Selective permeability of the BBB further limits the amount of therapeutic drug (antiretroviral) concentration reaching the CNS, thus leading to decreased drug efficacy and high dosing frequency and enhancing existing neurocognitive issues [297]. Magnetic nanocarriers loaded with antiretrovirals were shown to successfully cross the BBB without disrupting its integrity upon applying a non-invasive external magnetic field [298,299,300,301]. As the mechanisms of neurotoxicity mediated by both HIV infection and drugs of abuse are often similar, a drug antagonist that can block or mitigate the effects of drugs of abuse, in combination with an antiretroviral, can act as a promising therapeutic agent for polydrug users infected with HIV. Magnetic nanoformulation of nelfinavir (Nel) and rimcazole (Rico) are such examples. Rico is a potent sigma-1 (σ-1) receptor antagonist with a high affinity for dopamine transporters, hence possessing the capability to inhibit dopamine uptake. In combination with the protease inhibitor, Nelfinavir, Rico was successfully able to mitigate the effect of drugs of abuse on HIV infectivity, while being able to migrate across the BBB without disrupting its integrity and with minimum cell toxicity [302]. The pharmacologic interactions between antiretrovirals and drugs of abuse, as well as any other clinically prescribed or unprescribed drug, generally complicate the impacts of the seemingly attractive HIV treatment regimens [289,303].

## 9. Conclusions

Failure to eradicate the total viral load is a major limitation of ART. The limited accessibility of antiretrovirals into anatomical sanctuaries leads to viral persistence and the development of latent reservoirs. Despite the advances made in this field, HIV eradication is still a long way away. The focus should primarily be directed toward eliminating these latent reservoirs, as the viral particles existing in these reservoirs can contribute to the release of proinflammatory factors that contribute to the neurocognitive decline. Neurocognitive deficits occur in more than half of the people living with HIV-1/AIDS, even in those people where the infection is well controlled through ART. Thus, addressing CNS impairments in HIV patients is a priority.

The presence of comorbidities in HIV-infected individuals, the most common being drug abuse, contributes to the neurocognitive decline caused by the viral infection. Future research should be directed toward eliminating the neurocognitive symptoms associated with the comorbidities as well as latent reservoirs associated with HIV infection, especially in PWUDs. Stringent assessment of single and polydrug use on HIV infection, along with the consideration of ART regimen, must be made to understand their impact on CNS function. This necessitates the understanding of interactions among various drugs of abuse, drugs of abuse and HIV proteins, and drugs of abuse and ART drugs. Certain antiretrovirals may impair endothelial barrier integrity, contributing to increased BBB permeability. Studies need to be performed to characterize the combined effects of ART, HIV, and drugs of abuse on the integrity of BBB and their contribution to the development of HAND. Hence, there is an urgent need for the re-evaluation of the existing ART regimen in the context of neurocognitive impairment. The development of novel, targeted delivery of combination formulations, consisting of neuroprotective and antiretroviral drugs in conjunction with gene therapy, may thus prove attractive therapeutic interventions to eliminate HIV reservoirs and prevent neuro HIV.

## Figures and Tables

**Table 1:** Summary of similarities and differences between the mechanisms of neurotoxicity of major drugs of abuse.

Drug	Receptor Involved in Mediating Neurotoxicity	Neurotransmitter System Disrupted	Effect on Viral Proteins/HIV Replication	Mechanism of Neurotoxicity	Interaction with ART
Opioids	MOR: Mu (μ) opioid receptor KOR: Kappa (κ) opioid receptor DOR: Delta (δ) opioid receptor	Dopaminergic system	Potentiates viral replication through: Increased expression of galectin 1 Inhibition of interferon Increased expression of CCR5	Disrupt the integrity of BBB Oxidative stress Neuroinflammation through cytokine secretion	Compete with antiretrovirals for cytochrome P450 enzymes (CYP3A4, CYP2D6, CYP2C19, CYP2C9, and CYP2D67)
Cannabinoids	CB1 receptor CB2 receptor NMDA receptor	Endocannabinoid system Glutamatergic system	Gp120 increases the expression of FAAH, the enzyme that metabolizes the neuroprotective endocannabinoid, anandamide	Apoptosis Neuroinflammation	Compete with antiretrovirals for cytochrome P450 enzymes (CYP3A4 and CYP2C19)
Cocaine	Dopamine (Serotonin) (Norepinephrine)	Dopaminergic system	The combination of GP120 and cocaine increases the production of ROS and iNOS expression Cocaine augments Tat-mediated mitochondrial depolarization and production of ROS	Oxidative stress Epigenetic modifications Apoptosis	Compete with antiretrovirals for cytochrome P450 enzymes (CYP3A4 and CYP3A5)
Methamphetamine	Dopamine Serotonin Norepinephrine NMDA receptor	Dopaminergic system Glutamatergic system	Methamphetamine and Tat synergistically decrease dopamine reserves by binding to VMAT and DAT	Disrupt the integrity of BBB Impairing glial signaling Excitotoxicity	Compete with antiretrovirals for cytochrome P450 enzymes (CYP2D6 and CYP3A4)
Ethanol	NMDA receptor GABA receptor	Glutamatergic system GABAergic system	Stimulates HIV transcription through TNF secretion The combination of ethanol and Tat increases cytokine production	Apoptosis Ethanol exerts direct neurotoxicity	Compete with antiretrovirals for cytochrome P450 enzymes (CYP2E1 and CYP3A4)

## Data Availability

Not applicable.

## References

[1] UNAIDS. https://www.unaids.org/en/resources/documents/2019/2019-UNAIDS-data.

[2] Tyagi M., Romerio F. (2011). Models of HIV-1 persistence in the CD4+ T cell compartment: Past, present and future. Curr. HIV Res..

[3] Tyagi M., Bukrinsky M. (2012). Human immunodeficiency virus (HIV) latency: The major hurdle in HIV eradication. Mol. Med..

[4] Hokello J., Sharma A.L., Dimri M., Tyagi M. (2019). Insights into the HIV Latency and the Role of Cytokines. Pathogens.

[5] Sharma A.L., Hokello J., Sonti S., Zicari S., Sun L., Alqatawni A., Bukrinsky M., Simon G., Chauhan A., Daniel R. (2020). CBF-1 Promotes the Establishment and Maintenance of HIV Latency by Recruiting Polycomb Repressive Complexes, PRC1 and PRC2, at HIV LTR. Viruses.

[6] Zicari S., Sharma A.L., Sahu G., Dubrovsky L., Sun L., Yue H., Jada T., Ochem A., Simon G., Bukrinsky M. (2020). DNA dependent protein kinase (DNA-PK) enhances HIV transcription by promoting RNA polymerase II activity and recruitment of transcription machinery at HIV LTR. Oncotarget.

[7] Alqatawni A., Sharma A.L., Attilus B., Tyagi M., Daniel R. (2020). Shedding Light on the Role of Extracellular Vesicles in HIV Infection and Wound Healing. Viruses.

[8] Pomerantz R.J. (2003). Reservoirs, sanctuaries, and residual disease: The hiding spots of HIV-1. HIV Clin. Trials.

[9] Sonti S., Sharma A.L., Tyagi M. (2021). HIV-1 persistence in the CNS: Mechanisms of latency, pathogenesis and an update on eradication strategies. Virus Res..

[10] Tyagi M., Iordanskiy S., Ammosova T., Kumari N., Smith K., Breuer D., Ilatovskiy A.V., Kont Y.S., Ivanov A., Uren A. (2015). Reactivation of latent HIV-1 provirus via targeting protein phosphatase-1. Retrovirology.

[11] Hokello J., Sharma A.L., Tyagi M. (2020). Efficient Non-Epigenetic Activation of HIV Latency through the T-Cell Receptor Signalosome. Viruses.

[12] Hokello J., Lakhikumar Sharma A., Tyagi M. (2021). AP-1 and NF-kappaB synergize to transcriptionally activate latent HIV upon T-cell receptor activation. FEBS Lett..

[13] Hokello J., Sharma A.L., Tyagi M. (2021). Combinatorial Use of Both Epigenetic and Non-Epigenetic Mechanisms to Efficiently Reactivate HIV Latency. Int. J. Mol. Sci..

[14] Proust A., Barat C., Leboeuf M., Drouin J., Gagnon M.T., Vanasse F., Tremblay M.J. (2020). HIV-1 infection and latency-reversing agents bryostatin-1 and JQ1 disrupt amyloid beta homeostasis in human astrocytes. Glia.

[15] Lamers S.L., Salemi M., Galligan D.C., Morris A., Gray R., Fogel G., Zhao L., McGrath M.S. (2010). Human immunodeficiency virus-1 evolutionary patterns associated with pathogenic processes in the brain. J. Neurovirol..

[16] Borrajo Lopez A., Penedo M.A., Rivera-Baltanas T., Perez-Rodriguez D., Alonso-Crespo D., Fernandez-Pereira C., Olivares J.M., Agis-Balboa R.C. (2021). Microglia: The Real Foe in HIV-1-Associated Neurocognitive Disorders?. Biomedicines.

[17] Bougea A., Spantideas N., Galanis P., Gkekas G., Thomaides T. (2019). Optimal treatment of HIV-associated neurocognitive disorders: Myths and reality. A critical review. Ther. Adv. Infect. Dis..

[18] Wang Y., Liu M., Lu Q., Farrell M., Lappin J.M., Shi J., Lu L., Bao Y. (2020). Global prevalence and burden of HIV-associated neurocognitive disorder: A meta-analysis. Neurology.

[19] Underwood J., Robertson K.R., Winston A. (2015). Could antiretroviral neurotoxicity play a role in the pathogenesis of cognitive impairment in treated HIV disease?. AIDS.

[20] Hokello J., Sharma A.L., Tyagi P., Bhushan A., Tyagi M. (2021). Human Immunodeficiency Virus Type-1 (HIV-1) Transcriptional Regulation, Latency and Therapy in the Central Nervous System. Vaccines.

[21] Hokello J., Sharma A.L., Tyagi M. (2021). An Update on the HIV DNA Vaccine Strategy. Vaccines.

[22] Sil S., Niu F., Chivero E.T., Singh S., Periyasamy P., Buch S. (2020). Role of Inflammasomes in HIV-1 and Drug Abuse Mediated Neuroinflammaging. Cells.

[23] Sahu G., Farley K., El-Hage N., Aiamkitsumrit B., Fassnacht R., Kashanchi F., Ochem A., Simon G.L., Karn J., Hauser K.F. (2015). Cocaine promotes both initiation and elongation phase of HIV-1 transcription by activating NF-kappaB and MSK1 and inducing selective epigenetic modifications at HIV-1 LTR. Virology.

[24] Shapshak P., Duncan R., Nath A., Turchan J., Pandjassarame K., Rodriguez H., Duran E.M., Ziegler F., Amaro E., Lewis A. (2006). Gene chromosomal organization and expression in cultured human neurons exposed to cocaine and HIV-1 proteins gp120 and tat: Drug abuse and NeuroAIDS. Front. Biosci..

[25] Tyagi M., Bukrinsky M., Simon G.L. (2016). Mechanisms of HIV Transcriptional Regulation by Drugs of Abuse. Curr. HIV Res..

[26] Khan S.I., Reza M.M., Crowe S.M., Rahman M., Hellard M., Sarker M.S., Chowdhury E.I., Rana A., Sacks-Davis R., Banu S. (2019). People who inject drugs in Bangladesh—The untold burden!. Int. J. Infect. Dis..

[27] Kostaki E., Magiorkinis G., Psichogiou M., Flampouris A., Iliopoulos P., Papachristou E., Daikos G.L., Bonovas S., Otelea D., Friedman S.R. (2017). Detailed Molecular Surveillance of the HIV-1 Outbreak Among People who Inject Drugs (PWID) in Athens During a Period of Four Years. Curr. HIV Res..

[28] Meacham M.C., Rudolph A.E., Strathdee S.A., Rusch M.L., Brouwer K.C., Patterson T.L., Vera A., Rangel G., Roesch S.C. (2015). Polydrug Use and HIV Risk Among People Who Inject Heroin in Tijuana, Mexico: A Latent Class Analysis. Subst. Use Misuse.

[29] Niculescu I., Paraschiv S., Paraskevis D., Abagiu A., Batan I., Banica L., Otelea D. (2015). Recent HIV-1 Outbreak Among Intravenous Drug Users in Romania: Evidence for Cocirculation of CRF14_BG and Subtype F1 Strains. AIDS Res. Hum. Retrovir..

[30] Degenhardt L., Peacock A., Colledge S., Leung J., Grebely J., Vickerman P., Stone J., Cunningham E.B., Trickey A., Dumchev K. (2017). Global prevalence of injecting drug use and sociodemographic characteristics and prevalence of HIV, HBV, and HCV in people who inject drugs: A multistage systematic review. Lancet Glob. Health.

[31] CDC. https://www.cdc.gov/hiv/pdf/library/reports/surveillance/cdc-hiv-surveillance-report-2015-vol-27.pdf.

[32] Des Jarlais D.C., Kerr T., Carrieri P., Feelemyer J., Arasteh K. (2016). HIV infection among persons who inject drugs: Ending old epidemics and addressing new outbreaks. AIDS.

[33] Booth R.E., Watters J.K., Chitwood D.D. (1993). HIV risk-related sex behaviors among injection drug users, crack smokers, and injection drug users who smoke crack. Am. J. Public Health.

[34] Pechansky F., Woody G., Inciardi J., Surratt H., Kessler F., Von Diemen L., Bumaguin D.B. (2006). HIV seroprevalence among drug users: An analysis of selected variables based on 10 years of data collection in Porto Alegre, Brazil. Drug Alcohol Depend..

[35] Webber M.P., Schoenbaum E.E., Gourevitch M.N., Buono D., Klein R.S. (1999). A prospective study of HIV disease progression in female and male drug users. AIDS.

[36] Siddiqui N.S., Brown L.S., Makuch R.W. (1993). Short-term declines in CD4 levels associated with cocaine use in HIV-1 seropositive, minority injecting drug users. J. Natl. Med. Assoc..

[37] Arendt V., Guillorit L., Origer A., Sauvageot N., Vaillant M., Fischer A., Goedertz H., Francois J.H., Alexiev I., Staub T. (2019). Injection of cocaine is associated with a recent HIV outbreak in people who inject drugs in Luxembourg. PLoS ONE.

[38] Levine A.J., Reynolds S., Cox C., Miller E.N., Sinsheimer J.S., Becker J.T., Martin E., Sacktor N., Ned Sacktor for the Neuropsychology Working Group of the Multicenter AIDS Cohort Study (2014). The longitudinal and interactive effects of HIV status, stimulant use, and host genotype upon neurocognitive functioning. J. Neurovirol..

[39] Holtz T.H., Pattanasin S., Chonwattana W., Tongtoyai J., Chaikummao S., Varangrat A., Mock P.A. (2015). Longitudinal analysis of key HIV-risk behavior patterns and predictors in men who have sex with men, Bangkok, Thailand. Arch. Sex. Behav..

[40] Rosinska M., Sieroslawski J., Wiessing L. (2015). High regional variability of HIV, HCV and injecting risks among people who inject drugs in Poland: Comparing a cross-sectional bio-behavioural study with case-based surveillance. BMC Infect. Dis..

[41] Chang S.L., Connaghan K.P., Wei Y., Li M.D. (2014). NeuroHIV and use of addictive substances. Int. Rev. Neurobiol..

[42] Huang Y.F., Yang J.Y., Nelson K.E., Kuo H.S., Lew-Ting C.Y., Yang C.H., Chen C.H., Chang F.Y., Liu H.R. (2014). Changes in HIV incidence among people who inject drugs in Taiwan following introduction of a harm reduction program: A study of two cohorts. PLoS Med..

[43] Shiau S., Arpadi S.M., Yin M.T., Martins S.S. (2017). Patterns of drug use and HIV infection among adults in a nationally representative sample. Addict. Behav..

[44] Hartzler B., Dombrowski J.C., Crane H.M., Eron J.J., Geng E.H., Christopher Mathews W., Mayer K.H., Moore R.D., Mugavero M.J., Napravnik S. (2017). Prevalence and Predictors of Substance Use Disorders Among HIV Care Enrollees in the United States. AIDS Behav..

[45] Duko B., Ayalew M., Ayano G. (2019). The prevalence of alcohol use disorders among people living with HIV/AIDS: A systematic review and meta-analysis. Subst. Abuse Treat. Prev. Policy.

[46] Price R.W., Spudich S. (2008). Antiretroviral therapy and central nervous system HIV type 1 infection. J. Infect. Dis..

[47] Lanman T., Letendre S., Ma Q., Bang A., Ellis R. (2021). CNS Neurotoxicity of Antiretrovirals. J. Neuroimmune Pharmacol..

[48] Kohler J.J., Lewis W. (2007). A brief overview of mechanisms of mitochondrial toxicity from NRTIs. Environ. Mol. Mutagen.

[49] Nooka S., Ghorpade A. (2018). Organellar stress intersects the astrocyte endoplasmic reticulum, mitochondria and nucleolus in HIV associated neurodegeneration. Cell Death Dis..

[50] Hung K.M., Chen P.C., Hsieh H.C., Calkins M.J. (2017). Mitochondrial defects arise from nucleoside/nucleotide reverse transcriptase inhibitors in neurons: Potential contribution to HIV-associated neurocognitive disorders. Biochim. Biophys. Acta Mol. Basis Dis..

[51] Blas-Garcia A., Polo M., Alegre F., Funes H.A., Martinez E., Apostolova N., Esplugues J.V. (2014). Lack of mitochondrial toxicity of darunavir, raltegravir and rilpivirine in neurons and hepatocytes: A comparison with efavirenz. J. Antimicrob. Chemother..

[52] Ciavatta V.T., Bichler E.K., Speigel I.A., Elder C.C., Teng S.L., Tyor W.R., Garcia P.S. (2017). In vitro and Ex vivo Neurotoxic Effects of Efavirenz are Greater than Those of Other Common Antiretrovirals. Neurochem. Res..

[53] Lv Z., Chu Y., Wang Y. (2015). HIV protease inhibitors: A review of molecular selectivity and toxicity. HIV AIDS (Auckl.).

[54] Patel P., Louie S., Pai M., Kiser J., Gubbins P., Rodvold K. (2018). Drug Interactions in HIV: Protease and Integrase Inhibitors. Drug Interactions in Infectious Diseases: Antimicrobial Drug Interactions.

[55] Gray J., Young B. (2009). Acute onset insomnia associated with the initiation of raltegravir: A report of two cases and literature review. AIDS Patient Care STDS.

[56] Ellis R.J., Deutsch R., Heaton R.K., Marcotte T.D., McCutchan J.A., Nelson J.A., Abramson I., Thal L.J., Atkinson J.H., Wallace M.R. (1997). Neurocognitive impairment is an independent risk factor for death in HIV infection. San Diego HIV Neurobehavioral Research Center Group. Arch. Neurol..

[57] Heaton R.K., Franklin D.R., Ellis R.J., McCutchan J.A., Letendre S.L., Leblanc S., Corkran S.H., Duarte N.A., Clifford D.B., Woods S.P. (2011). HIV-associated neurocognitive disorders before and during the era of combination antiretroviral therapy: Differences in rates, nature, and predictors. J. Neurovirol..

[58] Gonzalez-Scarano F., Martin-Garcia J. (2005). The neuropathogenesis of AIDS. Nat. Rev. Immunol..

[59] Ketzler S., Weis S., Haug H., Budka H. (1990). Loss of neurons in the frontal cortex in AIDS brains. Acta Neuropathol..

[60] Reyes M.G., Faraldi F., Senseng C.S., Flowers C., Fariello R. (1991). Nigral degeneration in acquired immune deficiency syndrome (AIDS). Acta Neuropathol..

[61] Graus F., Ribalta T., Abos J., Alom J., Cruz-Sanchez F., Mallolas J., Miro J.M., Cardesa A., Tolosa E. (1990). Subacute cerebellar syndrome as the first manifestation of AIDS dementia complex. Acta Neurol. Scand..

[62] Sanchez A.B., Kaul M. (2017). Neuronal Stress and Injury Caused by HIV-1, cART and Drug Abuse: Converging Contributions to HAND. Brain Sci..

[63] Eisele E., Siliciano R.F. (2012). Redefining the viral reservoirs that prevent HIV-1 eradication. Immunity.

[64] Gray L.R., Roche M., Flynn J.K., Wesselingh S.L., Gorry P.R., Churchill M.J. (2014). Is the central nervous system a reservoir of HIV-1?. Curr. Opin. HIV AIDS.

[65] Cosenza M.A., Zhao M.L., Si Q., Lee S.C. (2002). Human brain parenchymal microglia express CD14 and CD45 and are productively infected by HIV-1 in HIV-1 encephalitis. Brain Pathol..

[66] Churchill M.J., Gorry P.R., Cowley D., Lal L., Sonza S., Purcell D.F., Thompson K.A., Gabuzda D., McArthur J.C., Pardo C.A. (2006). Use of laser capture microdissection to detect integrated HIV-1 DNA in macrophages and astrocytes from autopsy brain tissues. J. Neurovirol..

[67] Churchill M.J., Wesselingh S.L., Cowley D., Pardo C.A., McArthur J.C., Brew B.J., Gorry P.R. (2009). Extensive astrocyte infection is prominent in human immunodeficiency virus-associated dementia. Ann. Neurol..

[68] Gianella S., Kosakovsky Pond S.L., Oliveira M.F., Scheffler K., Strain M.C., De la Torre A., Letendre S., Smith D.M., Ellis R.J. (2016). Compartmentalized HIV rebound in the central nervous system after interruption of antiretroviral therapy. Virus Evol..

[69] Dahl V., Gisslen M., Hagberg L., Peterson J., Shao W., Spudich S., Price R.W., Palmer S. (2014). An example of genetically distinct HIV type 1 variants in cerebrospinal fluid and plasma during suppressive therapy. J. Infect. Dis..

[70] Sturdevant C.B., Dow A., Jabara C.B., Joseph S.B., Schnell G., Takamune N., Mallewa M., Heyderman R.S., Van Rie A., Swanstrom R. (2012). Central nervous system compartmentalization of HIV-1 subtype C variants early and late in infection in young children. PLoS Pathog..

[71] Meucci O., Fatatis A., Simen A.A., Bushell T.J., Gray P.W., Miller R.J. (1998). Chemokines regulate hippocampal neuronal signaling and gp120 neurotoxicity. Proc. Natl. Acad. Sci. USA.

[72] Piller S.C., Jans P., Gage P.W., Jans D.A. (1998). Extracellular HIV-1 virus protein R causes a large inward current and cell death in cultured hippocampal neurons: Implications for AIDS pathology. Proc. Natl. Acad. Sci. USA.

[73] Mattson M.P., Haughey N.J., Nath A. (2005). Cell death in HIV dementia. Cell Death Differ..

[74] Lannuzel A., Lledo P.M., Lamghitnia H.O., Vincent J.D., Tardieu M. (1995). HIV-1 envelope proteins gp120 and gp160 potentiate NMDA-induced [Ca^2+^]_i_ increase, alter [Ca^2+^]_i_ homeostasis and induce neurotoxicity in human embryonic neurons. Eur. J. Neurosci..

[75] Garden G.A., Guo W., Jayadev S., Tun C., Balcaitis S., Choi J., Montine T.J., Moller T., Morrison R.S. (2004). HIV associated neurodegeneration requires p53 in neurons and microglia. FASEB J..

[76] Medders K.E., Sejbuk N.E., Maung R., Desai M.K., Kaul M. (2010). Activation of p38 MAPK is required in monocytic and neuronal cells for HIV glycoprotein 120-induced neurotoxicity. J. Immunol..

[77] Garden G.A., Budd S.L., Tsai E., Hanson L., Kaul M., D’Emilia D.M., Friedlander R.M., Yuan J., Masliah E., Lipton S.A. (2002). Caspase cascades in human immunodeficiency virus-associated neurodegeneration. J. Neurosci..

[78] Jordan-Sciutto K.L., Wang G., Murphey-Corb M., Wiley C.A. (2002). Cell cycle proteins exhibit altered expression patterns in lentiviral-associated encephalitis. J. Neurosci..

[79] Haughey N.J., Cutler R.G., Tamara A., McArthur J.C., Vargas D.L., Pardo C.A., Turchan J., Nath A., Mattson M.P. (2004). Perturbation of sphingolipid metabolism and ceramide production in HIV-dementia. Ann. Neurol..

[80] Kaul M., Garden G.A., Lipton S.A. (2001). Pathways to neuronal injury and apoptosis in HIV-associated dementia. Nature.

[81] Genis P., Jett M., Bernton E.W., Boyle T., Gelbard H.A., Dzenko K., Keane R.W., Resnick L., Mizrachi Y., Volsky D.J. (1992). Cytokines and arachidonic metabolites produced during human immunodeficiency virus (HIV)-infected macrophage-astroglia interactions: Implications for the neuropathogenesis of HIV disease. J. Exp. Med..

[82] Bennett B.A., Rusyniak D.E., Hollingsworth C.K. (1995). HIV-1 gp120-induced neurotoxicity to midbrain dopamine cultures. Brain Res..

[83] Yeung M.C., Geertsma F., Liu J., Lau A.S. (1998). Inhibition of HIV-1 gp120-induced apoptosis in neuroblastoma SK-N-SH cells by an antisense oligodeoxynucleotide against p53. AIDS.

[84] Bagetta G., Corasaniti M.T., Paoletti A.M., Berliocchi L., Nistico R., Giammarioli A.M., Malorni W., Finazzi-Agro A. (1998). HIV-1 gp120-induced apoptosis in the rat neocortex involves enhanced expression of cyclo-oxygenase type 2 (COX-2). Biochem. Biophys. Res. Commun..

[85] O’Donnell L.A., Agrawal A., Jordan-Sciutto K.L., Dichter M.A., Lynch D.R., Kolson D.L. (2006). Human immunodeficiency virus (HIV)-induced neurotoxicity: Roles for the NMDA receptor subtypes. J. Neurosci..

[86] Corasaniti M.T., Strongoli M.C., Piccirilli S., Nistico R., Costa A., Bilotta A., Turano P., Finazzi-Agro A., Bagetta G. (2000). Apoptosis induced by gp120 in the neocortex of rat involves enhanced expression of cyclooxygenase type 2 and is prevented by NMDA receptor antagonists and by the 21-aminosteroid U-74389G. Biochem. Biophys. Res. Commun..

[87] Lipton S.A. (1994). Neuronal injury associated with HIV-1 and potential treatment with calcium-channel and NMDA antagonists. Dev. Neurosci..

[88] Doble A. (1999). The role of excitotoxicity in neurodegenerative disease: Implications for therapy. Pharmacol. Ther..

[89] Ohagen A., Ghosh S., He J., Huang K., Chen Y., Yuan M., Osathanondh R., Gartner S., Shi B., Shaw G. (1999). Apoptosis induced by infection of primary brain cultures with diverse human immunodeficiency virus type 1 isolates: Evidence for a role of the envelope. J. Virol..

[90] Raber J., Toggas S.M., Lee S., Bloom F.E., Epstein C.J., Mucke L. (1996). Central nervous system expression of HIV-1 Gp120 activates the hypothalamic-pituitary-adrenal axis: Evidence for involvement of NMDA receptors and nitric oxide synthase. Virology.

[91] Jana A., Pahan K. (2004). Human immunodeficiency virus type 1 gp120 induces apoptosis in human primary neurons through redox-regulated activation of neutral sphingomyelinase. J. Neurosci..

[92] Tyagi M., Pearson R.J., Karn J. (2010). Establishment of HIV latency in primary CD4+ cells is due to epigenetic transcriptional silencing and P-TEFb restriction. J. Virol..

[93] Kim Y.K., Bourgeois C.F., Pearson R., Tyagi M., West M.J., Wong J., Wu S.Y., Chiang C.M., Karn J. (2006). Recruitment of TFIIH to the HIV LTR is a rate-limiting step in the emergence of HIV from latency. EMBO J..

[94] Tyagi M., Karn J. (2007). CBF-1 promotes transcriptional silencing during the establishment of HIV-1 latency. EMBO J..

[95] Pearson R., Kim Y.K., Hokello J., Lassen K., Friedman J., Tyagi M., Karn J. (2008). Epigenetic silencing of human immunodeficiency virus (HIV) transcription by formation of restrictive chromatin structures at the viral long terminal repeat drives the progressive entry of HIV into latency. J. Virol..

[96] Marzio G., Tyagi M., Gutierrez M.I., Giacca M. (1998). HIV-1 tat transactivator recruits p300 and CREB-binding protein histone acetyltransferases to the viral promoter. Proc. Natl. Acad. Sci. USA.

[97] Kiernan R.E., Vanhulle C., Schiltz L., Adam E., Xiao H., Maudoux F., Calomme C., Burny A., Nakatani Y., Jeang K.T. (1999). HIV-1 tat transcriptional activity is regulated by acetylation. EMBO J..

[98] Marino J., Maubert M.E., Mele A.R., Spector C., Wigdahl B., Nonnemacher M.R. (2020). Functional impact of HIV-1 Tat on cells of the CNS and its role in HAND. Cell. Mol. Life Sci..

[99] Nath A., Psooy K., Martin C., Knudsen B., Magnuson D.S., Haughey N., Geiger J.D. (1996). Identification of a human immunodeficiency virus type 1 Tat epitope that is neuroexcitatory and neurotoxic. J. Virol..

[100] Song L., Nath A., Geiger J.D., Moore A., Hochman S. (2003). Human immunodeficiency virus type 1 Tat protein directly activates neuronal N-methyl-D-aspartate receptors at an allosteric zinc-sensitive site. J. Neurovirol..

[101] Li W., Huang Y., Reid R., Steiner J., Malpica-Llanos T., Darden T.A., Shankar S.K., Mahadevan A., Satishchandra P., Nath A. (2008). NMDA receptor activation by HIV-Tat protein is clade dependent. J. Neurosci..

[102] Bruce-Keller A.J., Chauhan A., Dimayuga F.O., Gee J., Keller J.N., Nath A. (2003). Synaptic transport of human immunodeficiency virus-Tat protein causes neurotoxicity and gliosis in rat brain. J. Neurosci..

[103] McManus C.M., Weidenheim K., Woodman S.E., Nunez J., Hesselgesser J., Nath A., Berman J.W. (2000). Chemokine and chemokine-receptor expression in human glial elements: Induction by the HIV protein, Tat, and chemokine autoregulation. Am. J. Pathol..

[104] Rappaport J., Joseph J., Croul S., Alexander G., Del Valle L., Amini S., Khalili K. (1999). Molecular pathway involved in HIV-1-induced CNS pathology: Role of viral regulatory protein, Tat. J. Leukoc. Biol..

[105] Pei L., Lee F.J., Moszczynska A., Vukusic B., Liu F. (2004). Regulation of dopamine D1 receptor function by physical interaction with the NMDA receptors. J. Neurosci..

[106] Missale C., Fiorentini C., Busi C., Collo G., Spano P.F. (2006). The NMDA/D1 receptor complex as a new target in drug development. Curr. Top. Med. Chem..

[107] Silvers J.M., Aksenova M.V., Aksenov M.Y., Mactutus C.F., Booze R.M. (2007). Neurotoxicity of HIV-1 Tat protein: Involvement of D1 dopamine receptor. Neurotoxicology.

[108] Aksenova M.V., Silvers J.M., Aksenov M.Y., Nath A., Ray P.D., Mactutus C.F., Booze R.M. (2006). HIV-1 Tat neurotoxicity in primary cultures of rat midbrain fetal neurons: Changes in dopamine transporter binding and immunoreactivity. Neurosci. Lett..

[109] Wallace D.R., Dodson S., Nath A., Booze R.M. (2006). Estrogen attenuates gp120- and tat1-72-induced oxidative stress and prevents loss of dopamine transporter function. Synapse.

[110] Aksenov M.Y., Hasselrot U., Bansal A.K., Wu G., Nath A., Anderson C., Mactutus C.F., Booze R.M. (2001). Oxidative damage induced by the injection of HIV-1 Tat protein in the rat striatum. Neurosci. Lett..

[111] Kruman I.I., Nath A., Mattson M.P. (1998). HIV-1 protein Tat induces apoptosis of hippocampal neurons by a mechanism involving caspase activation, calcium overload, and oxidative stress. Exp. Neurol..

[112] Haughey N.J., Holden C.P., Nath A., Geiger J.D. (1999). Involvement of inositol 1,4,5-trisphosphate-regulated stores of intracellular calcium in calcium dysregulation and neuron cell death caused by HIV-1 protein tat. J. Neurochem..

[113] New D.R., Maggirwar S.B., Epstein L.G., Dewhurst S., Gelbard H.A. (1998). HIV-1 Tat induces neuronal death via tumor necrosis factor-alpha and activation of non-N-methyl-D-aspartate receptors by a NFkappaB-independent mechanism. J. Biol. Chem..

[114] Polazzi E., Levi G., Minghetti L. (1999). Human immunodeficiency virus type 1 Tat protein stimulates inducible nitric oxide synthase expression and nitric oxide production in microglial cultures. J. Neuropathol. Exp. Neurol..

[115] Mohammad Ahmadi Soleimani S., Ekhtiari H., Cadet J.L. (2016). Drug-induced neurotoxicity in addiction medicine: From prevention to harm reduction. Prog. Brain Res..

[116] Frank M.G., Weber M.D., Watkins L.R., Maier S.F. (2015). Stress sounds the alarmin: The role of the danger-associated molecular pattern HMGB1 in stress-induced neuroinflammatory priming. Brain Behav. Immun..

[117] Salter M.W., Stevens B. (2017). Microglia emerge as central players in brain disease. Nat. Med..

[118] Bylicky M.A., Mueller G.P., Day R.M. (2018). Mechanisms of Endogenous Neuroprotective Effects of Astrocytes in Brain Injury. Oxid Med. Cell Longev..

[119] Hodes G.E., Kana V., Menard C., Merad M., Russo S.J. (2015). Neuroimmune mechanisms of depression. Nat. Neurosci..

[120] Felger J.C., Miller A.H. (2012). Cytokine effects on the basal ganglia and dopamine function: The subcortical source of inflammatory malaise. Front. Neuroendocrinol..

[121] Dong Y., Taylor J.R., Wolf M.E., Shaham Y. (2017). Circuit and Synaptic Plasticity Mechanisms of Drug Relapse. J. Neurosci..

[122] Birben E., Sahiner U.M., Sackesen C., Erzurum S., Kalayci O. (2012). Oxidative stress and antioxidant defense. World Allergy Organ. J..

[123] Lyras L., Cairns N.J., Jenner A., Jenner P., Halliwell B. (1997). An assessment of oxidative damage to proteins, lipids, and DNA in brain from patients with Alzheimer’s disease. J. Neurochem..

[124] Poli G., Leonarduzzi G., Biasi F., Chiarpotto E. (2004). Oxidative stress and cell signalling. Curr. Med. Chem..

[125] Bannon M.J. (2005). The dopamine transporter: Role in neurotoxicity and human disease. Toxicol. Appl. Pharmacol..

[126] Graham D.G., Tiffany S.M., Bell W.R., Gutknecht W.F. (1978). Autoxidation versus covalent binding of quinones as the mechanism of toxicity of dopamine, 6-hydroxydopamine, and related compounds toward C1300 neuroblastoma cells in vitro. Mol. Pharmacol..

[127] Lyles J., Cadet J.L. (2003). Methylenedioxymethamphetamine (MDMA, Ecstasy) neurotoxicity: Cellular and molecular mechanisms. Brain Res. Brain Res. Rev..

[128] Yamamoto B.K., Bankson M.G. (2005). Amphetamine neurotoxicity: Cause and consequence of oxidative stress. Crit. Rev. Neurobiol..

[129] Dietrich J.B., Mangeol A., Revel M.O., Burgun C., Aunis D., Zwiller J. (2005). Acute or repeated cocaine administration generates reactive oxygen species and induces antioxidant enzyme activity in dopaminergic rat brain structures. Neuropharmacology.

[130] Macedo D.S., de Vasconcelos S.M., dos Santos R.S., Aguiar L.M., Lima V.T., Viana G.S., de Sousa F.C. (2005). Cocaine alters catalase activity in prefrontal cortex and striatum of mice. Neurosci. Lett..

[131] Lipton J.W., Gyawali S., Borys E.D., Koprich J.B., Ptaszny M., McGuire S.O. (2003). Prenatal cocaine administration increases glutathione and alpha-tocopherol oxidation in fetal rat brain. Brain Res. Dev. Brain Res..

[132] Qiusheng Z., Yuntao Z., Rongliang Z., Dean G., Changling L. (2005). Effects of verbascoside and luteolin on oxidative damage in brain of heroin treated mice. Pharmazie.

[133] Xu B., Wang Z., Li G., Li B., Lin H., Zheng R., Zheng Q. (2006). Heroin-administered mice involved in oxidative stress and exogenous antioxidant-alleviated withdrawal syndrome. Basic Clin. Pharmacol. Toxicol..

[134] Alberts B., Johnson A., Lewis J., Raff M., Roberts K., Walter P. (2002). Programmed Cell Death (Apoptosis).

[135] Raychaudhuri S. (2010). A minimal model of signaling network elucidates cell-to-cell stochastic variability in apoptosis. PLoS ONE.

[136] Jayanthi S., Deng X., Ladenheim B., McCoy M.T., Cluster A., Cai N.S., Cadet J.L. (2005). Calcineurin/NFAT-induced up-regulation of the Fas ligand/Fas death pathway is involved in methamphetamine-induced neuronal apoptosis. Proc. Natl. Acad. Sci. USA.

[137] Krasnova I.N., Ladenheim B., Cadet J.L. (2005). Amphetamine induces apoptosis of medium spiny striatal projection neurons via the mitochondria-dependent pathway. FASEB J..

[138] Dey S., Mactutus C.F., Booze R.M., Snow D.M. (2007). Cocaine exposure in vitro induces apoptosis in fetal locus coeruleus neurons by altering the Bax/Bcl-2 ratio and through caspase-3 apoptotic signaling. Neuroscience.

[139] Imam S.Z., Duhart H.M., Skinner J.T., Ali S.F. (2005). Cocaine induces a differential dose-dependent alteration in the expression profile of immediate early genes, transcription factors, and caspases in PC12 cells: A possible mechanism of neurotoxic damage in cocaine addiction. Ann. N. Y. Acad. Sci..

[140] Poon H.F., Abdullah L., Mullan M.A., Mullan M.J., Crawford F.C. (2007). Cocaine-induced oxidative stress precedes cell death in human neuronal progenitor cells. Neurochem. Int..

[141] Cunha-Oliveira T., Rego A.C., Garrido J., Borges F., Macedo T., Oliveira C.R. (2007). Street heroin induces mitochondrial dysfunction and apoptosis in rat cortical neurons. J. Neurochem..

[142] Oliveira M.T., Rego A.C., Macedo T.R., Oliveira C.R. (2003). Drugs of abuse induce apoptotic features in PC12 cells. Ann. N. Y. Acad. Sci..

[143] Dong X.X., Wang Y., Qin Z.H. (2009). Molecular mechanisms of excitotoxicity and their relevance to pathogenesis of neurodegenerative diseases. Acta Pharmacol. Sin..

[144] Dani A., Huang B., Bergan J., Dulac C., Zhuang X. (2010). Superresolution imaging of chemical synapses in the brain. Neuron.

[145] Chávez-Castillo M., Rojas M., Bautista J. (2017). Excitotoxicity: An Organized Crime at The Cellular Level. Arch. Med..

[146] Bronner F. (2001). Extracellular and intracellular regulation of calcium homeostasis. Sci. World J..

[147] Pivovarova N.B., Andrews S.B. (2010). Calcium-dependent mitochondrial function and dysfunction in neurons. FEBS J..

[148] Watkins J.C., Jane D.E. (2006). The glutamate story. Br. J. Pharmacol..

[149] Novelli A., Reilly J.A., Lysko P.G., Henneberry R.C. (1988). Glutamate becomes neurotoxic via the N-methyl-D-aspartate receptor when intracellular energy levels are reduced. Brain Res..

[150] Mao J., Sung B., Ji R.R., Lim G. (2002). Neuronal apoptosis associated with morphine tolerance: Evidence for an opioid-induced neurotoxic mechanism. J. Neurosci..

[151] Reid M.S., Hsu K., Berger S.P. (1997). Cocaine and amphetamine preferentially stimulate glutamate release in the limbic system: Studies on the involvement of dopamine. Synapse.

[152] Williams J.M., Steketee J.D. (2004). Cocaine increases medial prefrontal cortical glutamate overflow in cocaine-sensitized rats: A time course study. Eur. J. Neurosci..

[153] Wolf M.E., Xue C.J., Li Y., Wavak D. (2000). Amphetamine increases glutamate efflux in the rat ventral tegmental area by a mechanism involving glutamate transporters and reactive oxygen species. J. Neurochem..

[154] Langlais P.J., Mair R.G. (1990). Protective effects of the glutamate antagonist MK-801 on pyrithiamine-induced lesions and amino acid changes in rat brain. J. Neurosci..

[155] Jaenisch R., Bird A. (2003). Epigenetic regulation of gene expression: How the genome integrates intrinsic and environmental signals. Nat. Genet..

[156] Nestler E.J., Luscher C. (2019). The Molecular Basis of Drug Addiction: Linking Epigenetic to Synaptic and Circuit Mechanisms. Neuron.

[157] Tyagi M., Weber J., Bukrinsky M., Simon G.L. (2016). The effects of cocaine on HIV transcription. J. Neurovirol..

[158] Koob G., Kreek M.J. (2007). Stress, dysregulation of drug reward pathways, and the transition to drug dependence. Am. J. Psychiatry.

[159] Kelley A.E., Berridge K.C. (2002). The neuroscience of natural rewards: Relevance to addictive drugs. J. Neurosci..

[160] Kumar A., Choi K.H., Renthal W., Tsankova N.M., Theobald D.E., Truong H.T., Russo S.J., Laplant Q., Sasaki T.S., Whistler K.N. (2005). Chromatin remodeling is a key mechanism underlying cocaine-induced plasticity in striatum. Neuron.

[161] Botia B., Legastelois R., Alaux-Cantin S., Naassila M. (2012). Expression of ethanol-induced behavioral sensitization is associated with alteration of chromatin remodeling in mice. PLoS ONE.

[162] Renthal W., Carle T.L., Maze I., Covington H.E., Truong H.T., Alibhai I., Kumar A., Montgomery R.L., Olson E.N., Nestler E.J. (2008). Delta FosB mediates epigenetic desensitization of the c-fos gene after chronic amphetamine exposure. J. Neurosci..

[163] Hamilton P.J., Lim C.J., Nestler E.J., Heller E.A. (2018). Neuroepigenetic Editing. Methods Mol. Biol..

[164] Heller E.A., Cates H.M., Pena C.J., Sun H., Shao N., Feng J., Golden S.A., Herman J.P., Walsh J.J., Mazei-Robison M. (2014). Locus-specific epigenetic remodeling controls addiction- and depression-related behaviors. Nat. Neurosci..

[165] Heller E.A., Hamilton P.J., Burek D.D., Lombroso S.I., Pena C.J., Neve R.L., Nestler E.J. (2016). Targeted Epigenetic Remodeling of the Cdk5 Gene in Nucleus Accumbens Regulates Cocaine- and Stress-Evoked Behavior. J. Neurosci..

[166] Kalant H., Kalant O.J. (1975). Death in amphetamine users: Causes and rates. Can. Med. Assoc. J..

[167] Sandoval V., Hanson G.R., Fleckenstein A.E. (2000). Methamphetamine decreases mouse striatal dopamine transporter activity: Roles of hyperthermia and dopamine. Eur. J. Pharmacol..

[168] Lin P.S., Quamo S., Ho K.C., Gladding J. (1991). Hyperthermia enhances the cytotoxic effects of reactive oxygen species to Chinese hamster cells and bovine endothelial cells in vitro. Radiat. Res..

[169] Jayanthi S., Deng X., Noailles P.A., Ladenheim B., Cadet J.L. (2004). Methamphetamine induces neuronal apoptosis via cross-talks between endoplasmic reticulum and mitochondria-dependent death cascades. FASEB J..

[170] Sharma H.S., Olsson Y., Dey P.K. (1990). Changes in blood-brain barrier and cerebral blood flow following elevation of circulating serotonin level in anesthetized rats. Brain Res..

[171] Sharma H.S. (2007). Methods to produce hyperthermia-induced brain dysfunction. Prog. Brain Res..

[172] Levy A.D., Baumann M.H., Van de Kar L.D. (1994). Monoaminergic regulation of neuroendocrine function and its modification by cocaine. Front. Neuroendocrinol..

[173] Cunningham K.A., Paris J.M., Goeders N.E. (1992). Chronic cocaine enhances serotonin autoregulation and serotonin uptake binding. Synapse.

[174] Bose J., Hedden S.L., Lipari R.N., Park-Lee E., Porter J.D., Pemberton M.R. (2016). Key Substance Use and Mental Health Indicators in the United States: Results from the 2017 National Survey on Drug Use and Health. Subst. Abus. Ment. Health Serv. Adm..

[175] Dutta R., Roy S. (2012). Mechanism(s) involved in opioid drug abuse modulation of HAND. Curr. HIV Res..

[176] Young A.M., Havens J.R. (2012). Transition from first illicit drug use to first injection drug use among rural Appalachian drug users: A cross-sectional comparison and retrospective survival analysis. Addiction.

[177] Mateu-Gelabert P., Guarino H., Jessell L., Teper A. (2015). Injection and sexual HIV/HCV risk behaviors associated with nonmedical use of prescription opioids among young adults in New York City. J. Subst. Abuse Treat..

[178] Cunningham C.O. (2018). Opioids and HIV Infection: From Pain Management to Addiction Treatment. Top. Antivir. Med..

[179] Weisberg D.F., Gordon K.S., Barry D.T., Becker W.C., Crystal S., Edelman E.J., Gaither J., Gordon A.J., Goulet J., Kerns R.D. (2015). Long-term Prescription of Opioids and/or Benzodiazepines and Mortality Among HIV-Infected and Uninfected Patients. J. Acquir. Immune Defic. Syndr..

[180] Filipczak-Bryniarska I., Nowak B., Sikora E., Nazimek K., Woron J., Wordliczek J., Bryniarski K. (2012). The influence of opioids on the humoral and cell-mediated immune responses in mice. The role of macrophages. Pharmacol. Rep..

[181] Dave R.S., Khalili K. (2010). Morphine treatment of human monocyte-derived macrophages induces differential miRNA and protein expression: Impact on inflammation and oxidative stress in the central nervous system. J. Cell Biochem..

[182] Reynolds J.L., Law W.C., Mahajan S.D., Aalinkeel R., Nair B., Sykes D.E., Mammen M.J., Yong K.T., Hui R., Prasad P.N. (2012). Morphine and galectin-1 modulate HIV-1 infection of human monocyte-derived macrophages. J. Immunol..

[183] Guo C.J., Li Y., Tian S., Wang X., Douglas S.D., Ho W.Z. (2002). Morphine enhances HIV infection of human blood mononuclear phagocytes through modulation of beta-chemokines and CCR5 receptor. J. Investig. Med..

[184] Kim S., Hahn Y.K., Podhaizer E.M., McLane V.D., Zou S., Hauser K.F., Knapp P.E. (2018). A central role for glial CCR5 in directing the neuropathological interactions of HIV-1 Tat and opiates. J. Neuroinflamm..

[185] Ozawa T., Nakagawa T., Shige K., Minami M., Satoh M. (2001). Changes in the expression of glial glutamate transporters in the rat brain accompanied with morphine dependence and naloxone-precipitated withdrawal. Brain Res..

[186] Rodriguez M., Lapierre J., Ojha C.R., Estrada-Bueno H., Dever S.M., Gewirtz D.A., Kashanchi F., El-Hage N. (2017). Importance of Autophagy in Mediating Human Immunodeficiency Virus (HIV) and Morphine-Induced Metabolic Dysfunction and Inflammation in Human Astrocytes. Viruses.

[187] El-Hage N., Bruce-Keller A.J., Yakovleva T., Bazov I., Bakalkin G., Knapp P.E., Hauser K.F. (2008). Morphine exacerbates HIV-1 Tat-induced cytokine production in astrocytes through convergent effects on [Ca(2+)](i), NF-kappaB trafficking and transcription. PLoS ONE.

[188] Mahajan S.D., Aalinkeel R., Sykes D.E., Reynolds J.L., Bindukumar B., Fernandez S.F., Chawda R., Shanahan T.C., Schwartz S.A. (2008). Tight junction regulation by morphine and HIV-1 tat modulates blood-brain barrier permeability. J. Clin. Immunol..

[189] Yousif S., Saubamea B., Cisternino S., Marie-Claire C., Dauchy S., Scherrmann J.M., Decleves X. (2008). Effect of chronic exposure to morphine on the rat blood-brain barrier: Focus on the P-glycoprotein. J. Neurochem..

[190] Coley J.S., Calderon T.M., Gaskill P.J., Eugenin E.A., Berman J.W. (2015). Dopamine increases CD14+CD16+ monocyte migration and adhesion in the context of substance abuse and HIV neuropathogenesis. PLoS ONE.

[191] Calderon T.M., Williams D.W., Lopez L., Eugenin E.A., Cheney L., Gaskill P.J., Veenstra M., Anastos K., Morgello S., Berman J.W. (2017). Dopamine Increases CD14(+)CD16(+) Monocyte Transmigration across the Blood Brain Barrier: Implications for Substance Abuse and HIV Neuropathogenesis. J. Neuroimmune Pharmacol..

[192] Gaskill P.J., Calderon T.M., Luers A.J., Eugenin E.A., Javitch J.A., Berman J.W. (2009). Human immunodeficiency virus (HIV) infection of human macrophages is increased by dopamine: A bridge between HIV-associated neurologic disorders and drug abuse. Am. J. Pathol..

[193] Gaskill P.J., Yano H.H., Kalpana G.V., Javitch J.A., Berman J.W. (2014). Dopamine receptor activation increases HIV entry into primary human macrophages. PLoS ONE.

[194] Iuvone T., Capasso A., D’Acquisto F., Carnuccio R. (1995). Opioids inhibit the induction of nitric oxide synthase in J774 macrophages. Biochem. Biophys. Res. Commun..

[195] Turchan-Cholewo J., Dimayuga F.O., Gupta S., Keller J.N., Knapp P.E., Hauser K.F., Bruce-Keller A.J. (2009). Morphine and HIV-Tat increase microglial-free radical production and oxidative stress: Possible role in cytokine regulation. J. Neurochem..

[196] Riss G.L., Chang D.I., Wevers C., Westendorf A.M., Buer J., Scherbaum N., Hansen W. (2012). Opioid maintenance therapy restores CD4+ T cell function by normalizing CD4+CD25(high) regulatory T cell frequencies in heroin user. Brain Behav. Immun..

[197] Sharp B.M., McAllen K., Gekker G., Shahabi N.A., Peterson P.K. (2001). Immunofluorescence detection of delta opioid receptors (DOR) on human peripheral blood CD4+ T cells and DOR-dependent suppression of HIV-1 expression. J. Immunol..

[198] Peterson P.K., Gekker G., Lokensgard J.R., Bidlack J.M., Chang A.C., Fang X., Portoghese P.S. (2001). Kappa-opioid receptor agonist suppression of HIV-1 expression in CD4+ lymphocytes. Biochem. Pharmacol..

[199] Plein L.M., Rittner H.L. (2018). Opioids and the immune system—Friend or foe. Br. J. Pharmacol..

[200] Desai N., Burns L., Gong Y., Zhi K., Kumar A., Summers N., Kumar S., Cory T.J. (2020). An update on drug-drug interactions between antiretroviral therapies and drugs of abuse in HIV systems. Expert Opin. Drug Metab. Toxicol..

[201] Feng X.Q., Zhu L.L., Zhou Q. (2017). Opioid analgesics-related pharmacokinetic drug interactions: From the perspectives of evidence based on randomized controlled trials and clinical risk management. J. Pain Res..

[202] Wynn G.H., Cozza K.L., Zapor M.J., Wortmann G.W., Armstrong S.C. (2005). Med-psych drug-drug interactions update. Antiretrovirals, part III: Antiretrovirals and drugs of abuse. Psychosomatics.

[203] Carliner H., Brown Q.L., Sarvet A.L., Hasin D.S. (2017). Cannabis use, attitudes, and legal status in the U.S.: A review. Prev. Med..

[204] Lorenzetti V., Lubman D.I., Whittle S., Solowij N., Yucel M. (2010). Structural MRI findings in long-term cannabis users: What do we know?. Subst. Use Misuse.

[205] Cristiani S.A., Pukay-Martin N.D., Bornstein R.A. (2004). Marijuana use and cognitive function in HIV-infected people. J. Neuropsychiatry Clin. Neurosci..

[206] Kim H.J., Shin A.H., Thayer S.A. (2011). Activation of cannabinoid type 2 receptors inhibits HIV-1 envelope glycoprotein gp120-induced synapse loss. Mol. Pharmacol..

[207] Yi Y., Lee C., Liu Q.H., Freedman B.D., Collman R.G. (2004). Chemokine receptor utilization and macrophage signaling by human immunodeficiency virus type 1 gp120: Implications for neuropathogenesis. J. Neurovirol..

[208] Galve-Roperh I., Aguado T., Palazuelos J., Guzman M. (2008). Mechanisms of control of neuron survival by the endocannabinoid system. Curr. Pharm. Des..

[209] Benito C., Kim W.K., Chavarria I., Hillard C.J., Mackie K., Tolon R.M., Williams K., Romero J. (2005). A glial endogenous cannabinoid system is upregulated in the brains of macaques with simian immunodeficiency virus-induced encephalitis. J. Neurosci..

[210] Cosenza-Nashat M.A., Bauman A., Zhao M.L., Morgello S., Suh H.S., Lee S.C. (2011). Cannabinoid receptor expression in HIV encephalitis and HIV-associated neuropathologic comorbidities. Neuropathol. Appl. Neurobiol..

[211] Klegeris A., Bissonnette C.J., McGeer P.L. (2003). Reduction of human monocytic cell neurotoxicity and cytokine secretion by ligands of the cannabinoid-type CB2 receptor. Br. J. Pharmacol..

[212] Avraham H.K., Jiang S., Fu Y., Rockenstein E., Makriyannis A., Zvonok A., Masliah E., Avraham S. (2014). The cannabinoid CB(2) receptor agonist AM1241 enhances neurogenesis in GFAP/Gp120 transgenic mice displaying deficits in neurogenesis. Br. J. Pharmacol..

[213] Guzman M., Sanchez C., Galve-Roperh I. (2002). Cannabinoids and cell fate. Pharmacol. Ther..

[214] Mechoulam R., Panikashvili D., Shohami E. (2002). Cannabinoids and brain injury: Therapeutic implications. Trends Mol. Med..

[215] Minagar A., Shapshak P., Fujimura R., Ownby R., Heyes M., Eisdorfer C. (2002). The role of macrophage/microglia and astrocytes in the pathogenesis of three neurologic disorders: HIV-associated dementia, Alzheimer disease, and multiple sclerosis. J. Neurol. Sci..

[216] Weber A., Ni J., Ling K.H., Acheampong A., Tang-Liu D.D., Burk R., Cravatt B.F., Woodward D. (2004). Formation of prostamides from anandamide in FAAH knockout mice analyzed by HPLC with tandem mass spectrometry. J. Lipid Res..

[217] Maccarrone M., Piccirilli S., Battista N., Del Duca C., Nappi G., Corasaniti M.T., Finazzi-Agro A., Bagetta G. (2004). Enhanced anandamide degradation is associated with neuronal apoptosis induced by the HIV-1 coat glycoprotein gp120 in the rat neocortex. J. Neurochem..

[218] Howlett A.C., Barth F., Bonner T.I., Cabral G., Casellas P., Devane W.A., Felder C.C., Herkenham M., Mackie K., Martin B.R. (2002). International Union of Pharmacology. XXVII. Classification of cannabinoid receptors. Pharmacol. Rev..

[219] Ranganathan M., D’Souza D.C. (2006). The acute effects of cannabinoids on memory in humans: A review. Psychopharmacology.

[220] Volkow N.D., Baler R.D., Compton W.M., Weiss S.R. (2014). Adverse health effects of marijuana use. N. Engl. J. Med..

[221] Lucas C.J., Galettis P., Schneider J. (2018). The pharmacokinetics and the pharmacodynamics of cannabinoids. Br. J. Clin. Pharmacol..

[222] Kosel B.W., Aweeka F.T., Benowitz N.L., Shade S.B., Hilton J.F., Lizak P.S., Abrams D.I. (2002). The effects of cannabinoids on the pharmacokinetics of indinavir and nelfinavir. AIDS.

[223] Gannon B.M., Reichard E.E., Fantegrossi W.E. (2014). Psychostimulant Abuse and HIV Infection: Cocaine, methamphetamine, and “bath salts” cathinone analogues. Curr. Addict. Rep..

[224] Fardin S., Gholami S., Werner T., Kenney T., Silverman R., Metzger D., Alavi A., Tebas P. (2016). Deteriorating effects of cocaine abuse on brain metabolic function of HIV infected patients. J. Nucl. Med..

[225] Baldwin G.C., Tashkin D.P., Buckley D.M., Park A.N., Dubinett S.M., Roth M.D. (1997). Marijuana and cocaine impair alveolar macrophage function and cytokine production. Am. J. Respir. Crit. Care Med..

[226] Cao L., Walker M.P., Vaidya N.K., Fu M., Kumar S., Kumar A. (2016). Cocaine-Mediated Autophagy in Astrocytes Involves Sigma 1 Receptor, PI3K, mTOR, Atg5/7, Beclin-1 and Induces Type II Programed Cell Death. Mol. Neurobiol..

[227] Peterson P.K., Gekker G., Chao C.C., Schut R., Molitor T.W., Balfour H.H. (1991). Cocaine potentiates HIV-1 replication in human peripheral blood mononuclear cell cocultures. Involvement of transforming growth factor-beta. J. Immunol..

[228] Dhillon N.K., Williams R., Peng F., Tsai Y.J., Dhillon S., Nicolay B., Gadgil M., Kumar A., Buch S.J. (2007). Cocaine-mediated enhancement of virus replication in macrophages: Implications for human immunodeficiency virus-associated dementia. J. Neurovirol..

[229] Nath A., Maragos W.F., Avison M.J., Schmitt F.A., Berger J.R. (2001). Acceleration of HIV dementia with methamphetamine and cocaine. J. Neurovirol..

[230] Roth M.D., Tashkin D.P., Choi R., Jamieson B.D., Zack J.A., Baldwin G.C. (2002). Cocaine enhances human immunodeficiency virus replication in a model of severe combined immunodeficient mice implanted with human peripheral blood leukocytes. J. Infect. Dis..

[231] Swepson C., Ranjan A., Balasubramaniam M., Pandhare J., Dash C. (2016). Cocaine Enhances HIV-1 Transcription in Macrophages by Inducing p38 MAPK Phosphorylation. Front. Microbiol..

[232] Roth M.D., Whittaker K.M., Choi R., Tashkin D.P., Baldwin G.C. (2005). Cocaine and sigma-1 receptors modulate HIV infection, chemokine receptors, and the HPA axis in the huPBL-SCID model. J. Leukoc. Biol..

[233] Nair M.P., Mahajan S.D., Schwartz S.A., Reynolds J., Whitney R., Bernstein Z., Chawda R.P., Sykes D., Hewitt R., Hsiao C.B. (2005). Cocaine modulates dendritic cell-specific C type intercellular adhesion molecule-3-grabbing nonintegrin expression by dendritic cells in HIV-1 patients. J. Immunol..

[234] Turchan J., Anderson C., Hauser K.F., Sun Q., Zhang J., Liu Y., Wise P.M., Kruman I., Maragos W., Mattson M.P. (2001). Estrogen protects against the synergistic toxicity by HIV proteins, methamphetamine and cocaine. BMC Neurosci..

[235] Nath A., Hauser K.F., Wojna V., Booze R.M., Maragos W., Prendergast M., Cass W., Turchan J.T. (2002). Molecular basis for interactions of HIV and drugs of abuse. J. Acquir. Immune Defic. Syndr..

[236] Koutsilieri E., Gotz M.E., Sopper S., Sauer U., Demuth M., ter Meulen V., Riederer P. (1997). Regulation of glutathione and cell toxicity following exposure to neurotropic substances and human immunodeficiency virus-1 in vitro. J. Neurovirol..

[237] Nath A., Anderson C., Jones M., Maragos W., Booze R., Mactutus C., Bell J., Hauser K.F., Mattson M. (2000). Neurotoxicity and dysfunction of dopaminergic systems associated with AIDS dementia. J. Psychopharmacol..

[238] Yao H., Allen J.E., Zhu X., Callen S., Buch S. (2009). Cocaine and human immunodeficiency virus type 1 gp120 mediate neurotoxicity through overlapping signaling pathways. J. Neurovirol..

[239] Aksenov M.Y., Aksenova M.V., Nath A., Ray P.D., Mactutus C.F., Booze R.M. (2006). Cocaine-mediated enhancement of Tat toxicity in rat hippocampal cell cultures: The role of oxidative stress and D1 dopamine receptor. Neurotoxicology.

[240] Doke M., Jeganathan V., McLaughlin J.P., Samikkannu T. (2020). HIV-1 Tat and cocaine impact mitochondrial epigenetics: Effects on DNA methylation. Epigenetics.

[241] Yao H., Bethel-Brown C., Buch S. (2009). Cocaine Exposure Results in Formation of Dendritic Varicosity in Rat Primary Hippocampal Neurons. Am. J. Infect. Dis..

[242] Bagetta G., Piccirilli S., Del Duca C., Morrone L.A., Rombola L., Nappi G., De Alba J., Knowles R.G., Corasaniti M.T. (2004). Inducible nitric oxide synthase is involved in the mechanisms of cocaine enhanced neuronal apoptosis induced by HIV-1 gp120 in the neocortex of rat. Neurosci. Lett..

[243] Mohseni Ahooyi T., Shekarabi M., Torkzaban B., Langford T.D., Burdo T.H., Gordon J., Datta P.K., Amini S., Khalili K. (2018). Dysregulation of Neuronal Cholesterol Homeostasis upon Exposure to HIV-1 Tat and Cocaine Revealed by RNA-Sequencing. Sci. Rep..

[244] Lukas S.E., Sholar M., Lundahl L.H., Lamas X., Kouri E., Wines J.D., Kragie L., Mendelson J.H. (1996). Sex differences in plasma cocaine levels and subjective effects after acute cocaine administration in human volunteers. Psychopharmacology.

[245] Cone E.J. (1995). Pharmacokinetics and pharmacodynamics of cocaine. J. Anal Toxicol..

[246] Yang X., Wang Y., Li Q., Zhong Y., Chen L., Du Y., He J., Liao L., Xiong K., Yi C.X. (2018). The Main Molecular Mechanisms Underlying Methamphetamine- Induced Neurotoxicity and Implications for Pharmacological Treatment. Front. Mol. Neurosci..

[247] The L. (2018). Opioids and methamphetamine: A tale of two crises. Lancet.

[248] Zuckerman M.D., Boyer E.W. (2012). HIV and club drugs in emerging adulthood. Curr. Opin. Pediatr..

[249] Cadet J.L., Krasnova I.N. (2009). Molecular bases of methamphetamine-induced neurodegeneration. Int. Rev. Neurobiol..

[250] Cadet J.L., Bisagno V., Milroy C.M. (2014). Neuropathology of substance use disorders. Acta Neuropathol..

[251] Buttner A. (2011). Review: The neuropathology of drug abuse. Neuropathol Appl. Neurobiol..

[252] Theodore S., Cass W.A., Nath A., Maragos W.F. (2007). Progress in understanding basal ganglia dysfunction as a common target for methamphetamine abuse and HIV-1 neurodegeneration. Curr. HIV Res..

[253] Nath A. (2010). Human immunodeficiency virus-associated neurocognitive disorder: Pathophysiology in relation to drug addiction. Ann. N. Y. Acad. Sci..

[254] Kaushal N., Matsumoto R.R. (2011). Role of sigma receptors in methamphetamine-induced neurotoxicity. Curr. Neuropharmacol..

[255] Quinton M.S., Yamamoto B.K. (2006). Causes and consequences of methamphetamine and MDMA toxicity. AAPS J..

[256] Theodore S., Cass W.A., Maragos W.F. (2006). Methamphetamine and human immunodeficiency virus protein Tat synergize to destroy dopaminergic terminals in the rat striatum. Neuroscience.

[257] Miyatake M., Narita M., Shibasaki M., Nakamura A., Suzuki T. (2005). Glutamatergic neurotransmission and protein kinase C play a role in neuron-glia communication during the development of methamphetamine-induced psychological dependence. Eur. J. Neurosci..

[258] Hardingham G.E., Fukunaga Y., Bading H. (2002). Extrasynaptic NMDARs oppose synaptic NMDARs by triggering CREB shut-off and cell death pathways. Nat. Neurosci..

[259] Czub S., Koutsilieri E., Sopper S., Czub M., Stahl-Hennig C., Muller J.G., Pedersen V., Gsell W., Heeney J.L., Gerlach M. (2001). Enhancement of central nervous system pathology in early simian immunodeficiency virus infection by dopaminergic drugs. Acta Neuropathol..

[260] Hu S., Sheng W.S., Lokensgard J.R., Peterson P.K., Rock R.B. (2009). Preferential sensitivity of human dopaminergic neurons to gp120-induced oxidative damage. J. Neurovirol..

[261] Maragos W.F., Young K.L., Turchan J.T., Guseva M., Pauly J.R., Nath A., Cass W.A. (2002). Human immunodeficiency virus-1 Tat protein and methamphetamine interact synergistically to impair striatal dopaminergic function. J. Neurochem..

[262] Theodore S., Cass W.A., Dwoskin L.P., Maragos W.F. (2012). HIV-1 protein Tat inhibits vesicular monoamine transporter-2 activity in rat striatum. Synapse.

[263] Zhu J., Ananthan S., Mactutus C.F., Booze R.M. (2011). Recombinant human immunodeficiency virus-1 transactivator of transcription1-86 allosterically modulates dopamine transporter activity. Synapse.

[264] Alvarez-Carbonell D., Ye F., Ramanath N., Garcia-Mesa Y., Knapp P.E., Hauser K.F., Karn J. (2019). Cross-talk between microglia and neurons regulates HIV latency. PLoS Pathog..

[265] Pal D., Kwatra D., Minocha M., Paturi D.K., Budda B., Mitra A.K. (2011). Efflux transporters- and cytochrome P-450-mediated interactions between drugs of abuse and antiretrovirals. Life Sci..

[266] Nookala A.R., Li J., Ande A., Wang L., Vaidya N.K., Li W., Kumar S., Kumar A. (2016). Effect of Methamphetamine on Spectral Binding, Ligand Docking and Metabolism of Anti-HIV Drugs with CYP3A4. PLoS ONE.

[267] Ikonomidou C., Bittigau P., Ishimaru M.J., Wozniak D.F., Koch C., Genz K., Price M.T., Stefovska V., Horster F., Tenkova T. (2000). Ethanol-induced apoptotic neurodegeneration and fetal alcohol syndrome. Science.

[268] Pawlak R., Skrzypiec A., Sulkowski S., Buczko W. (2002). Ethanol-induced neurotoxicity is counterbalanced by increased cell proliferation in mouse dentate gyrus. Neurosci. Lett..

[269] Ikegami Y., Goodenough S., Inoue Y., Dodd P.R., Wilce P.A., Matsumoto I. (2003). Increased TUNEL positive cells in human alcoholic brains. Neurosci. Lett..

[270] Dingle G.A., Oei T.P. (1997). Is alcohol a cofactor of HIV and AIDS? Evidence from immunological and behavioral studies. Psychol. Bull..

[271] Meyerhoff D.J. (2001). Effects of alcohol and HIV infection on the central nervous system. Alcohol Res. Health.

[272] Friedman H., Newton C., Klein T.W. (2003). Microbial infections, immunomodulation, and drugs of abuse. Clin. Microbiol. Rev..

[273] Alak J.I., Shahbazian M., Huang D.S., Wang Y., Darban H., Jenkins E.M., Watson R.R. (1993). Alcohol and murine acquired immunodeficiency syndrome suppression of resistance to Cryptosporidium parvum infection during modulation of cytokine production. Alcohol. Clin. Exp. Res..

[274] Sepulveda R.T., Jiang S., Besselsen D.G., Watson R.R. (2002). Alcohol consumption during murine acquired immunodeficiency syndrome accentuates heart pathology due to coxsackievirus. Alcohol Alcohol..

[275] Acheampong E., Mukhtar M., Parveen Z., Ngoubilly N., Ahmad N., Patel C., Pomerantz R.J. (2002). Ethanol strongly potentiates apoptosis induced by HIV-1 proteins in primary human brain microvascular endothelial cells. Virology.

[276] Dong Q., Kelkar S., Xiao Y., Joshi-Barve S., McClain C.J., Barve S.S. (2000). Ethanol enhances TNF-alpha-inducible NFkappaB activation and HIV-1-LTR transcription in CD4+ Jurkat T lymphocytes. J. Lab. Clin. Med..

[277] Chen H., George I., Sperber K. (1998). Effect of ethanol on monocytic function in human immunodeficiency virus type 1 infection. Clin. Diagn. Lab. Immunol..

[278] Chen W., Tang Z., Fortina P., Patel P., Addya S., Surrey S., Acheampong E.A., Mukhtar M., Pomerantz R.J. (2005). Ethanol potentiates HIV-1 gp120-induced apoptosis in human neurons via both the death receptor and NMDA receptor pathways. Virology.

[279] Prakash O., Zhang P., Xie M., Ali M., Zhou P., Coleman R., Stoltz D.A., Bagby G.J., Shellito J.E., Nelson S. (1998). The human immunodeficiency virus type I Tat protein potentiates ethanol-induced neutrophil functional impairment in transgenic mice. Alcohol. Clin. Exp. Res..

[280] Marra C.M., Zhao Y., Clifford D.B., Letendre S., Evans S., Henry K., Ellis R.J., Rodriguez B., Coombs R.W., Schifitto G. (2009). Impact of combination antiretroviral therapy on cerebrospinal fluid HIV RNA and neurocognitive performance. AIDS.

[281] Smurzynski M., Wu K., Letendre S., Robertson K., Bosch R.J., Clifford D.B., Evans S., Collier A.C., Taylor M., Ellis R. (2011). Effects of central nervous system antiretroviral penetration on cognitive functioning in the ALLRT cohort. AIDS.

[282] Vittinghoff E., Hessol N.A., Bacchetti P., Fusaro R.E., Holmberg S.D., Buchbinder S.P. (2001). Cofactors for HIV disease progression in a cohort of homosexual and bisexual men. J. Acquir. Immune Defic. Syndr..

[283] Baum M.K., Rafie C., Lai S., Sales S., Page B., Campa A. (2009). Crack-cocaine use accelerates HIV disease progression in a cohort of HIV-positive drug users. J. Acquir. Immune Defic. Syndr..

[284] Hinkin C.H., Barclay T.R., Castellon S.A., Levine A.J., Durvasula R.S., Marion S.D., Myers H.F., Longshore D. (2007). Drug use and medication adherence among HIV-1 infected individuals. AIDS Behav..

[285] Hicks P.L., Mulvey K.P., Chander G., Fleishman J.A., Josephs J.S., Korthuis P.T., Hellinger J., Gaist P., Gebo K.A., Network H.I.V.R. (2007). The impact of illicit drug use and substance abuse treatment on adherence to HAART. AIDS Care.

[286] Meyer M.R., Maurer H.H. (2011). Absorption, distribution, metabolism and excretion pharmacogenomics of drugs of abuse. Pharmacogenomics.

[287] Anzenbacher P., Anzenbacherova E. (2001). Cytochromes P450 and metabolism of xenobiotics. Cell. Mol. Life Sci..

[288] Walubo A. (2007). The role of cytochrome P450 in antiretroviral drug interactions. Expert Opin. Drug Metab. Toxicol..

[289] Kumar S., Rao P.S., Earla R., Kumar A. (2015). Drug-drug interactions between anti-retroviral therapies and drugs of abuse in HIV systems. Expert Opin. Drug Metab. Toxicol..

[290] Lucas G.M., Griswold M., Gebo K.A., Keruly J., Chaisson R.E., Moore R.D. (2006). Illicit drug use and HIV-1 disease progression: A longitudinal study in the era of highly active antiretroviral therapy. Am. J. Epidemiol..

[291] Ferris M.J., Mactutus C.F., Booze R.M. (2008). Neurotoxic profiles of HIV, psychostimulant drugs of abuse, and their concerted effect on the brain: Current status of dopamine system vulnerability in NeuroAIDS. Neurosci. Biobehav. Rev..

[292] Ciccarone D. (2011). Stimulant abuse: Pharmacology, cocaine, methamphetamine, treatment, attempts at pharmacotherapy. Prim. Care.

[293] Borgmann K., Ghorpade A. (2015). HIV-1, methamphetamine and astrocytes at neuroinflammatory Crossroads. Front. Microbiol..

[294] Ramesh G., MacLean A.G., Philipp M.T. (2013). Cytokines and chemokines at the crossroads of neuroinflammation, neurodegeneration, and neuropathic pain. Mediators Inflamm..

[295] Atluri V.S., Hidalgo M., Samikkannu T., Kurapati K.R., Jayant R.D., Sagar V., Nair M.P. (2015). Effect of human immunodeficiency virus on blood-brain barrier integrity and function: An update. Front. Cell Neurosci..

[296] Stoops W.W., Rush C.R. (2013). Agonist replacement for stimulant dependence: A review of clinical research. Curr. Pharm. Des..

[297] Ingersoll K.S., Cohen J. (2008). The impact of medication regimen factors on adherence to chronic treatment: A review of literature. J. Behav. Med..

[298] Atluri V.S., Jayant R.D., Pilakka-Kanthikeel S., Garcia G., Samikkannu T., Yndart A., Kaushik A., Nair M. (2016). Development of TIMP1 magnetic nanoformulation for regulation of synaptic plasticity in HIV-1 infection. Int. J. Nanomed..

[299] Ding H., Sagar V., Agudelo M., Pilakka-Kanthikeel S., Atluri V.S., Raymond A., Samikkannu T., Nair M.P. (2014). Enhanced blood-brain barrier transmigration using a novel transferrin embedded fluorescent magneto-liposome nanoformulation. Nanotechnology.

[300] Jayant R.D., Atluri V.S., Agudelo M., Sagar V., Kaushik A., Nair M. (2015). Sustained-release nanoART formulation for the treatment of neuroAIDS. Int. J. Nanomed..

[301] Kaushik A., Jayant R.D., Nikkhah-Moshaie R., Bhardwaj V., Roy U., Huang Z., Ruiz A., Yndart A., Atluri V., El-Hage N. (2016). Magnetically guided central nervous system delivery and toxicity evaluation of magneto-electric nanocarriers. Sci. Rep..

[302] Jayant R.D., Atluri V.S.R., Tiwari S., Pilakka-Kanthikeel S., Kaushik A., Yndart A., Nair M. (2017). Novel nanoformulation to mitigate co-effects of drugs of abuse and HIV-1 infection: Towards the treatment of NeuroAIDS. J. Neurovirol..

[303] Stolbach A., Paziana K., Heverling H., Pham P. (2015). A Review of the Toxicity of HIV Medications II: Interactions with Drugs and Complementary and Alternative Medicine Products. J. Med. Toxicol..

